# Seeing is believing: Genome editing made easy with *RUBY* for educational purposes

**DOI:** 10.1093/plphys/kiaf265

**Published:** 2025-07-23

**Authors:** William K Sexton, Yanhao Cheng, Bing Yang, Hemayet Ullah, Yiping Qi, Shunyuan Xiao

**Affiliations:** Institute for Bioscience and Biotechnology Research, University of Maryland, Rockville, MD 20850, USA; Department of Plant Science and Landscape Architecture, University of Maryland, College Park, MD 20742, USA; Division of Plant Science and Technology, Bond Life Sciences Center, University of Missouri, Columbia, MO 65211, USA; Donald Danforth Plant Science Center, St. Louis, MO 63132, USA; Department of Biology, Howard University, Washington, DC 20059, USA; Institute for Bioscience and Biotechnology Research, University of Maryland, Rockville, MD 20850, USA; Department of Plant Science and Landscape Architecture, University of Maryland, College Park, MD 20742, USA; Institute for Bioscience and Biotechnology Research, University of Maryland, Rockville, MD 20850, USA; Department of Plant Science and Landscape Architecture, University of Maryland, College Park, MD 20742, USA

Dear Editor,

CRISPR-enabled genome-editing technologies have gained widespread application across diverse fields of research and engineering, particularly in agriculture (Wang and Doudna 2023; Pacesa et al. 2024). These advancements have captivated students from K-12 through college, sparking interest regardless of their specialization in bioscience or biotechnology. While CRISPR experiments have become routine in specialized biology laboratories, designing a CRISPR experiment module for high school or college biology courses faces challenges due to technical complexities and time constraints. Supported by an NSF collaborative grant entitled “RESEARCH-PGR: Genome-wide Quest for Non-Host Resistance Mechanisms in Plants,” we developed 2 straightforward CRISPR modules focused on editing visually scorable reporter genes, *RUBY* and *GFP*. These 2 modules were successfully implemented during 2 consecutive summer CRISPR workshops at Howard University, designed to train underrepresented minority undergraduate students. We envision that the 2 CRISPR modules, along with the experimental materials and protocols we developed, will serve as a valuable resource for high school science teachers and college biology instructors to incorporate into their curricula. To support this goal, we present the details of our work here, facilitating the dissemination of research protocols and related materials to the research and education community.

To ensure success of students with no prior laboratory experience in the 5-day workshop, we designed a “Seeing Is Believing—CRISPR Made Easy” educational program consisting of 3 mutually supportive components: (ⅰ) lectures on foundational knowledge, (ⅱ) hands-on practice with key experimental steps, and (ⅲ) analysis and reporting of results. Recognizing that gene-editing experiments are the primary time constraint, we developed 2 streamlined CRISPR strategies: one employing CRISPR/Cas9-mediated base editing to correct a nonsense mutation in *RUBY* (He et al. 2020) and the other utilizing CRISPR/Cas9 to perform targeted-mutagenesis of *GFP*. To expedite completion and enable visualization of successful gene editing, the respective DNA constructs were transiently expressed in either wild-type (WT) *Nicotiana benthamiana* or *N. benthamiana* stably expressing *GFP*.

The *RUBY* reporter is a synthetic open reading frame encoding 3 enzymes needed to produce the pigment betalain from tyrosine, which is readily available in plant cells (He et al. 2020). The 3 enzymes—P450 oxygenase CYP76AD1, L-DOPA 4,5-dioxygenase, and a glucosyltransferase—are translationally in-frame linked by sequences encoding the self-cleaving 2A peptides (He et al. 2020). Expression of *RUBY* in plant tissue produces a vivid red color visible to the naked eye, making it an excellent reporter in a classroom setting.

To demonstrate the power of CRISPR-enabled precise base editing, we attempted to use CRISPR base editing to revert a premature stop codon in *CYP76AD1,* which encodes the first enzyme in the RUBY pathway. We first identified 4 regions of *CYP76AD1* containing CAG or CAA within the 4th to 8th window at the 5′ end of a 20 nucleotide protospacer (upstream of an NGG or NGN PAM) suitable for serving as a small guide (sg) RNA binding site (Fig. 1A). We then introduced C → T mutations into these sites via site-directed mutagenesis to create early STOP codons generating 4 *RUBY* mutants in total (Table 1). We then separately inserted three 20 bp DNA fragments corresponding to spacers of the sgRNAs using NGG PAMs into an all-in-one expressing vector (pLR3850) containing the expression cassettes for the sgRNA and the Cas9 nickase (nCas9^D10A^) translationally fused with the engineered adenosine deaminase ecTadA8e (Supplementary Sequence S1), resulting in 3 functional adenine base editors (ABEs) for editing the mutated sequences (Fig. 1A; Table 1) (Lapinaite et al. 2020; Richter et al. 2020; Ren et al. 2021; Yan et al. 2021). The ABE utilizing the NGN PAM was constructed by inserting the relevant sgRNA and the base editor nzCas9-NG-ABE8e into the destination vector pCGS710 (Supplementary Sequence S2). We introduced the vectors containing the *RUBY* WT construct, the 4 *RUBY* mutant constructs, and the 4 ABE constructs into *Agrobacterium tumefaciens* strain GV3101 for a total of 9 strains. Agroinfiltration was used to transiently express WT *RUBY*, each *RUBY* mutant alone, or each *RUBY* mutant together with its corresponding *ABE* in leaves of *N. benthamiana*. To optimize expression, *Agrobacterium* containing the DNA construct expressing the coat protein (CP) of Turnip crinkle virus (TCV) (TCV-CP; GenBank ID: DQ286887) in the binary vector pPZP212 (GenBank ID: U10462.1) was also added to the agrobacterial mixture to suppress transgene silencing (Qu et al. 2003). At 2 days post-agroinfiltration (dpa), leaf areas infiltrated with WT *RUBY* turned a light red-purple color, while no sign of red color was observed in the tissue infiltrated with any other tested constructs. However, at 3 dpa, a faint red color could be seen in leaf tissue infiltrated with *RUBY-mutant#2* + *ABE*#*2* (Fig. 1B). Additionally, at 5 dpa, the areas agroinfiltrated with *RUBY-mutant#*2 + *ABE*#2 displayed a vivid red color, although not as strong as those with WT *RUBY* (Fig. 1B). Notably, the leaf areas agroinfiltrated with the other 3 *RUBY-mutant* + *ABE* (*#1*, *#3*, and *#4*) combinations also exhibited red color, albeit with lower intensity, whereas the areas infiltrated with the *RUBY* mutant constructs alone failed to produce any red pigment (Fig. 1B). The differences in editing efficiency among the ABEs, as reported by the slower and weaker betalain pigmentation observed with ABE#1, #3, and #4 compared with ABE#2, are likely due to variations in the respective local sequence contexts each of the 4 ABE acts on. If necessary, such variability in RUBY activity can be quantified by measuring betalain concentration in the infiltrated tissue (Pramanik et al. 2024). These results indicate that all the 4 ABEs must have worked to correct the corresponding nonsense mutations and that the pair *RUBY-mutant#2* + *ABE#2* outperformed the other 3 pairs. We thus selected the *RUBY-mutant#*2 + *ABE*#2 pair for the workshop.

**Figure 1.**
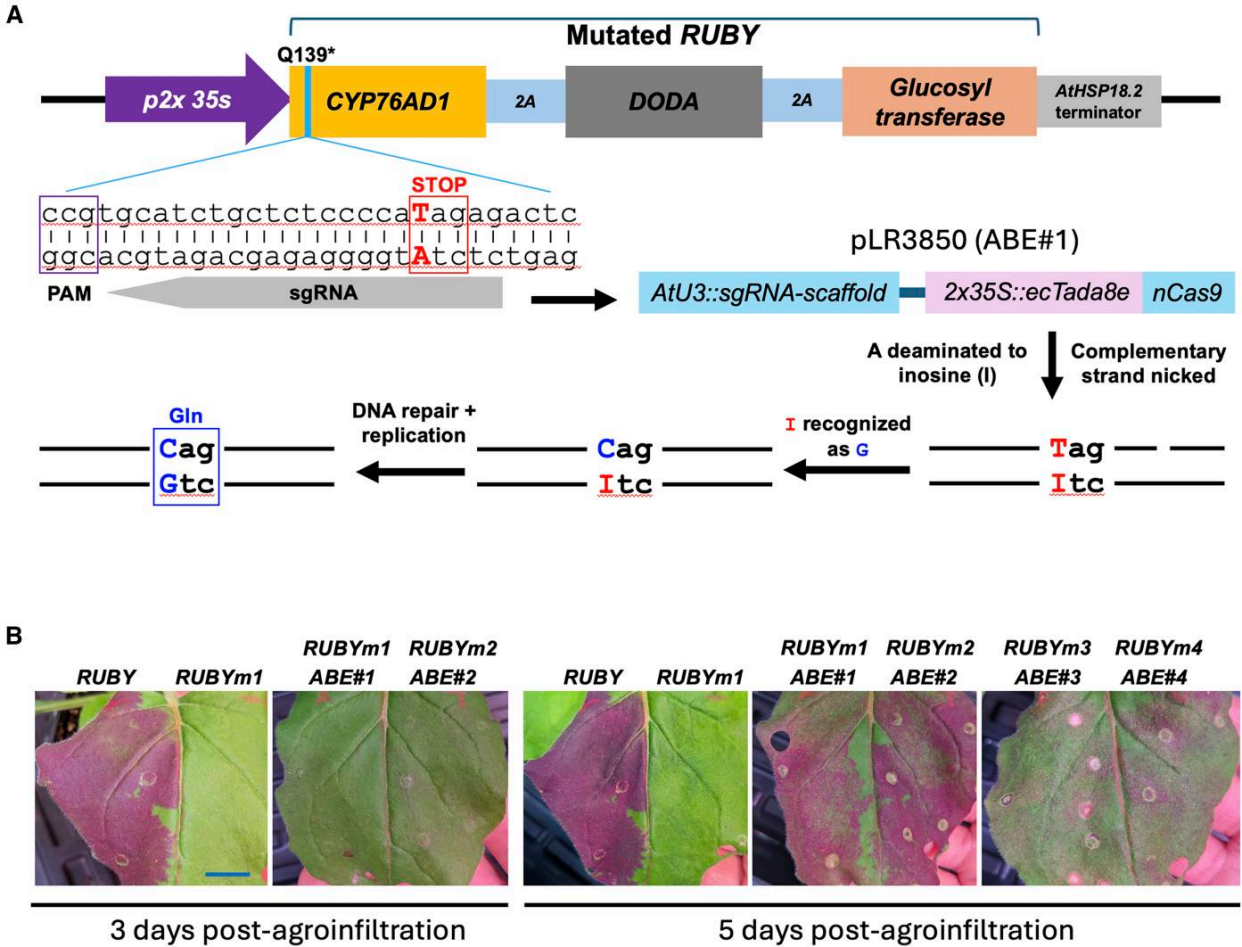
CRISPR/Cas9-enabled base editing of a nonsense mutation of *RUBY* in *N. benthamiana*. **A)** Schematic illustration of the experimental design for correcting a nonsense mutation (415TAG → 415CAG) in *RUBY* mutant 2 (*RUBYm2*) by a corresponding adenine base editor (ABE#2, Table 1). **B)** Leaf photos demonstrating successful correction of 4 nonsense mutations in *CYP76AD1* by their corresponding ABEs (Table 1) as reported by appearance of red color in the agroinfiltrated areas. Note, ABE#2 exhibited the best editing performance. Bar = 2 cm, which is applicable to all photos. ABE, adenine base editor; PAM, the protospacer adjacent motif.

**Table 1.** A compilation of nonfunctional RUBY mutants along with their corresponding CRISPR ABE constructs, sgRNA sequences, and respective editing efficiencies

Mutant ID	DNA mutation	Protein mutation	RUBY-ABE ID	sgRNA sequence with PAM (5′-3′)	RUBY activity reporting editing efficiency^a^
*RUBY*-Mutant #1	76**C**AG → 76**T**AG	Q26 to stop	#1_pYPQ5236	ctgctAggagaacagcagct**tga**	+++
*RUBY*-Mutant #2	415**C**AG → 415**T**AG	Q139 to stop	#2_pYPQ5237	tctAtggggagagcagatgca**cgg**	++++
*RUBY*-Mutant #3	604**C**AA → 604**T**AA	Q201 to stop	#3_pYPQ5238	cttAgctggtatgggacttg**tgg**	+
*RUBY*-Mutant #4	844**C**AG → 844**T**AG	Q282 to stop	#4_pYPQ5239	cgttctActtgaacagctgg**agg**	++
WT *RUBY*	NA	NA	NA	NA	++++++++

^a^The “+” symbols represent the intensity of red coloration in the infiltrated leaf tissue, as shown in Fig. 1B, providing a visual proxy for assessing the editing efficiency of the 4 RUBY-ABEs.

To showcase the ability of CRISPR-Cas9 to induce targeted mutagenesis, we designed sgRNAs specifically targeting the *GFP* gene stably expressed from the *35S* promoter in the *N. benthamiana* transgenic line 16c (Ruiz et al. 1998). We designed 4 sgRNAs to target different sequences in *GFP* to increase the probability of creating a large deletion (∼200 bp) (Fig. 2A). These sgRNAs were cloned into the binary vector pDGE463 which contains an intron-optimized Cas9 (zCas9i) under control of a 2*×35S* promoter (Stuttmann et al. 2021). The T-DNA also contains 2*×RFP* under control of the Arabidopsis (*Arabidopsis thaliana*) *2S3* promoter (Kroj et al. 2003), which allows for confirmation of successful transformation and expression of the transgenes.

**Figure 2.**
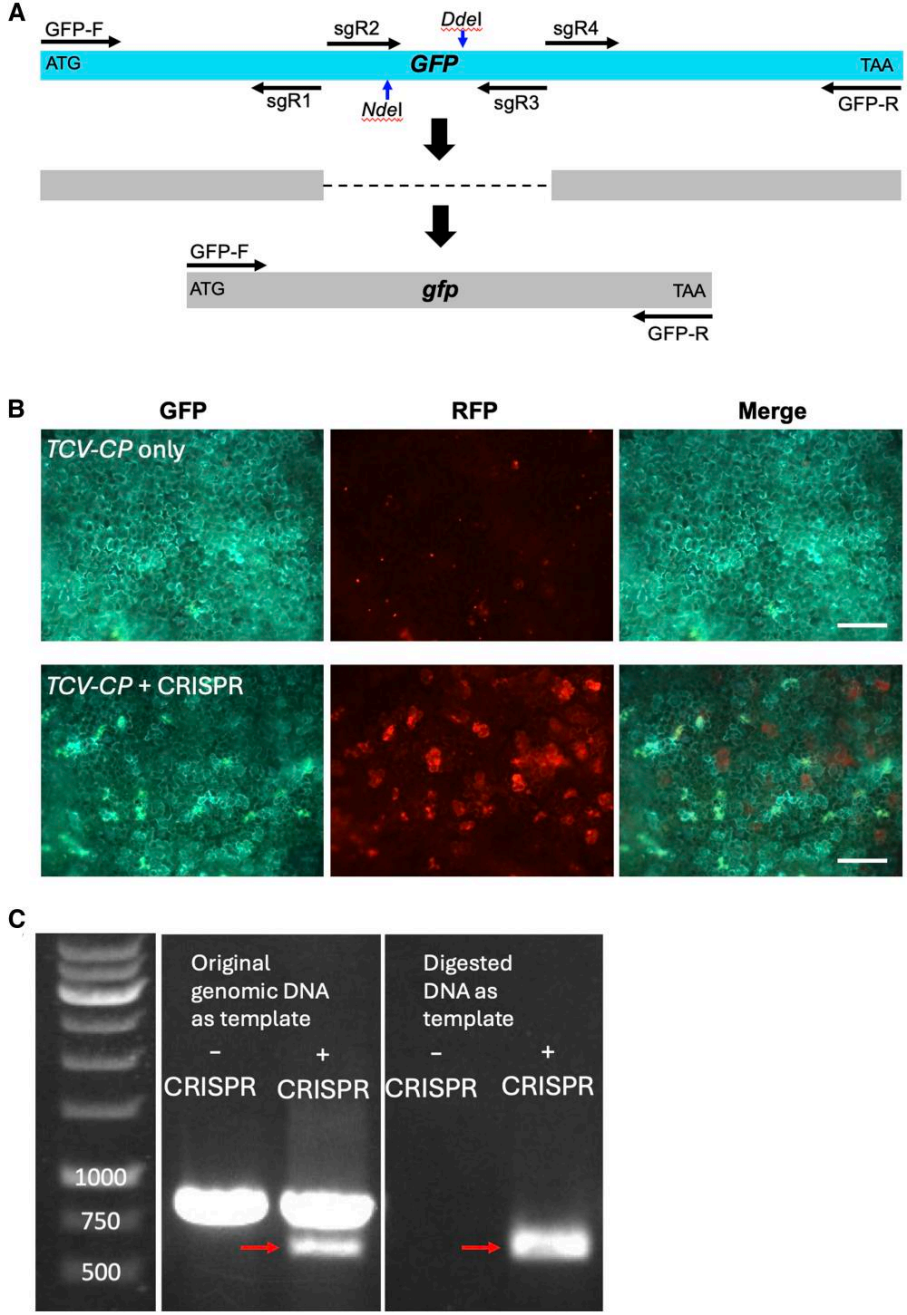
CRISPR/Cas9-targeted mutagenesis of *GFP* in *N. benthamiana*. **A)** Design of 4 sgRNAs. Note, *Nde*I and *Dde*I recognition sites are flanked by the sgRNA sites. **B)** Fluorescent images showing reduced GFP levels in leaf tissues agroinfiltrated with the CRISPR/Cas9 construct. **C)** Gel photos showing PCR amplification of the WT *GFP* and mutated *GFP* with large deletions ( arrows). Bar = 200 *µ*m. *TCV-CP*, the DNA construct expressing the capsid protein (CP) of Turnip crinkle virus (TCV).

We introduced the multiplex CRISPR vector targeting *GFP* into *Agrobacterium* strain GV3101 which was then infiltrated into leaves of *N. benthamiana* 16c along with *Agrobacterium* carrying *TCV-CP*. Leaves agroinfiltrated with *TCV-CP* alone served as control. At 3 or 4 dpa, while RFP signal was clearly detected, using an epifluorescent microscope, in leaf tissues agroinfiltrated with the multiplex CRISPR construct, indicating successful transformation, those tissues generally exhibited noticeable lower GFP levels compared with control tissue (Fig. 2B), suggesting active *GFP* mutagenesis. However, the reduction of GFP fluorescence in the leaf areas agroinfiltrated with the CRISPR construct may vary from case to case, making it challenging to consistently detect the difference by microscopy. To definitively determine if the *GFP* transgene is successfully mutated in some transformed leaf cells, we performed PCR with original genomic DNA or enrichment PCR with *Nde*I*/Dde*I-digested genomic DNA extracted from the agroinfiltrated leaf tissues using primers to amplify the *GFP* gene. Given that the *Nde*I and *Dde*I recognition sites are flanked by sgRNA#1- and sgRNA#4-binding sites, the digested genomic DNA would remove the WT *GFP* DNA thereby enriching amplification of mutant *GFP* DNA. Gel electrophoresis of the PCR products showed that while only the WT *GFP* fragment (∼800 bp) was amplified from DNA of the leaf tissues agroinfiltrated with *TCV-CP*, both the WT *GFP* band and a weak smaller band (∼600 bp) were detectable from DNA of the leaf tissues agroinfiltrated with the CRISPR construct (Fig. 2C). Notably, only a ∼600 bp fragment was amplified from the *Nde*I*/Dde*I-digested DNA prepared from tissues agroinfiltrated with the CRISPR construct (Fig. 2C). Sanger sequencing of the purified PCR products and subsequent sequence alignment, if time permits, can be added to this experiment to further help students understand the mechanisms of CRISPR-targeted mutagenesis as discussed in the CRISPR lecture. Together, these results indicate that our CRISPR-targeted mutagenesis of *GFP*, evidenced by reduced GFP levels and presence of a mutated *GFP* PCR product with a ∼200 bp internal deletion, was successful and could be deployed in the 5-day long workshop.

We prepared cultures of all required *Agrobacterium* strains, plants of *N. benthamiana* WT and the 16c transgenic line, and other necessary reagents and devices before the workshop (Supplementary Table S1). To ensure success of unexperienced students participating in the workshop, we developed detailed step-by-step protocols for these 2 CRISPR gene-editing strategies (Supplementary Information S1 to S3). To maximize students’ hand-on learning experience while overcoming time constraints, we re-arranged the order of specific experiments (e.g. conducting agroinfiltration of CRISPR constructs on day 1) and developed a detailed daily workshop schedule for lectures and experiments (Supplementary Information S4). We successfully ran 2 consecutive workshops at Howard University in 2023 and 2024 for 11 and 18 undergraduate students, respectively (Supplementary Fig. S1). Through agroinfiltration, all student groups (4–6 people each) succeeded in getting positive results with base editing of the *RUBY* mutant (Supplementary Fig. S2A and B). However, only about half of the groups obtained positive results with targeted mutagenesis of *GFP* (Supplementary Fig. S2C), likely because any mistake in DNA extraction, PCR, and/or gel electrophoresis could result in failure of amplifying even the WT *GFP* gene. Nevertheless, the failed experiments stimulated the students to think critically and troubleshoot. All students enjoyed the lectures on genome editing technologies given by the principal investigators and felt excited to “see” (literally by their naked eyes) CRISPR worked in their own hands (Supplementary Video S1). In addition, they also practiced fundamental molecular biology techniques (e.g. PCR, DNA ligation and cloning with plasmids, and *E. coli* transfection) and learned how to use the cloud-based biology research platform Benchling (https://www.benchling.com) to analyze DNA sequences and design sgRNAs. In the final day of the workshop, we held a Q&A session and an open discussion on the future development of CRISPR technologies to conclude the workshop.

In summary, we developed 2 CRISPR modules for training students who are interested in gaining hands-on experience with genome editing technologies in plants. These 2 CRISPR modules, combined with relevant lectures, provided students with a comprehensive understanding of gain-of-function and loss-of-function in genetics. The base-editing module for correcting a defective *RUBY* has been successfully demonstrated in an educational setting, achieving a 100% success rate with relatively modest resources. This makes it well-suited for use in biology lab courses and similar workshops. The success of this module, along with those described in a few recent studies, demonstrates RUBY's utility as a versatile visual reporter for various biological processes including RNA silencing (Tabara et al. 2024), pre-mRNA splicing (Prasad et al. 2024) and protein-protein interaction (Chen et al. 2023). All the biological materials created for the 2 CRISPR modules will be made available to the research and education community at request. The plasmids have been deposited to Addgene (RUBYm2: #232175, RUBY-ABE2, #232176) and the *Agrobacterium* and *E coli* strains have been deposited to the Arabidopsis Biological Resource Center (Agrobacterium strains: CD3-2957 for RUBYm2, CD3-2958 for RUBY-ABE2; CD3-2959 for zCas9i-4sgRNA-GFP/pDGE463; *E coli* strains: CD3-2960 for RUBYm2, CD3-2961 for RUBY-ABE2). The use of these teaching materials will not only promote the education of CRISPR-based genome editing tools but also spark students’ interest in plant biotechnology and research.

## Supplementary Material

kiaf265_Supplementary_Data

## Data Availability

The data supporting this work are available in the manuscript and presented in the Supplementary Information files (Supplementary Sequence S1 and S2, Supplementary Figure S1 and S2, Supplementary Table S1, Supplementary Video S1, and Supplementary Information S1 to S4). The plasmids were deposited to Addgene and the *Agrobacterium* and *E coli* strains were deposited to the Arabidopsis Biological Resource Center.

## Supplementary data

The following materials are available in the online version of this article.

**Supplementary Sequence S1.** DNA sequence of the all-in-one expressing vector pLR3850 used for making RUBY-ABEs.

**Supplementary Sequence S2.** The DNA sequence of the binary vector pCGS710 provided by Drs. Daniel Voytas and Colby Starker.

**Supplementary Figure S1.** Students’ success with CRISPR/Cas9-mediated gene editing.

**Supplementary Figure S2.** Group photos of the undergraduate students who participated in the 2 summer CRISPR workshops.

**Supplementary Table S1.** Plant materials, agrobacterial strains, primers and other key reagents for the CRISPR workshop

**Supplementary Video S1.** A short interview featuring students who participated in the 2023 Summer CRISPR Workshop.

**Supplementary Information S1.** Protocol #1: Transient gene expression in *N. benthamiana* via agroinfiltration.

**Supplementary Information S2.** Protocol #2: CRISPR-mediated base editing of a defective *RUBY* in *N. benthamiana*.

**Supplementary Information S3.** Protocol #3: Detection of CRISPR-induced deletions via PCR.

**Supplementary Information S4.** A daily schedule for a five-day CRISPR workshop.
