# Supplementary material for: Seeing is believing: Genome editing made easy with RUBY for educational purposes
Source: Plant Physiol. 2025 Jul 23;198(3):kiaf265. doi: 10.1093/plphys/kiaf265 (PMC12283223; doi:10.1093/plphys/kiaf265)
Supplement: kiaf265_Supplementary_Data [file kiaf265_supplementary_data.zip › Supplemental figures,table and other information revised.pdf]

Supplementary Sequence S1. DNA sequence of the all-in-one expressing vector pLR3850 used for making RUBY-ABEs.

CCAGCCAGCCAACAGCTCCCCGACCGGCAGCTCGGCACAAAATCACCACTCGATACAGGCAGCCCATCAGTCCGGGACGGCGTCAGCGGGAGAGCCG
TTGTAAGGCGGCAGACTTTGCTCATGTTACCGATGCTATTCGGAAGAACGGCAACTAAGCTGCCGGGTTTGAAACACGGATGATCTCGCGGAGGGTA
GCATGTTGATTGTAACGATGACAGAGCGTTGCTGCCTGTGATCACCGCGGTTTCAAAATCGGCTCCGTCGATACTATGTTATACGCCAACTTTGAAA
ACAACTTTGAAAAAGCTGTTTTCTGGTATTTAAGGTTTTAGAATGCAAGGAACAGTGAATTGGAGTTCGTCTTGTTATAATTAGCTTCTTGGGGTAT
CTTTAAATACTGTAGAAAAGAGGAAGGAAATAATAAATGGCTAAAATGAGAATATCACCGGAATTGAAAAAACTGATCGAAAAATACCGCTGCGTAA
AAGATACGGAAGGAATGTCTCCTGCTAAGGTATATAAGCTGGTGGGAGAAAATGAAAACCTATATTTAAAAATGACGGACAGCCGGTATAAAGGGAC
CACCTATGATGTGGAACGGGAAAAGGACATGATGCTATGGCTGGAAGGAAAGCTGCCTGTTCCAAAGGTCCTGCACTTTGAACGGCATGATGGCTGG
AGCAATCTGCTCATGAGTGAGGCCGATGGCGTCCTTTGCTCGGAAGAGTATGAAGATGAACAAAGCCCTGAAAAGATTATCGAGCTGTATGCGGAGT
GCATCAGGCTCTTTCACTCCATCGACATATCGGATTGTCCCTATACGAATAGCTTAGACAGCCGCTTAGCCGAATTGGATTACTTACTGAATAACGA
TCTGGCCGATGTGGATTGCGAAAACTGGGAAGAAGACACTCCATTTAAAGATCCGCGCGAGCTGTATGATTTTTTAAAGACGGAAAAGCCCGAAGAG
GAACTTGTCTTTTCCCACGGCGACCTGGGAGACAGCAACATCTTTGTGAAAGATGGCAAAGTAAGTGGCTTTATTGATCTTGGGAGAAGCGGCAGGG
CGGACAAGTGGTATGACATTGCCTTCTGCGTCCGGTCGATCAGGGAGGATATCGGGGAAGAACAGTATGTCGAGCTATTTTTTGACTTACTGGGGAT
CAAGCCTGATTGGGAGAAAATAAAATATTATATTTTACTGGATGAATTGTTTTAGTACCTAGAATGCATGACCAAAATCCCTTAACGTGAGTTTTCG
TTCCACTGAGCGTCAGACCCCGTAGAAAAGATCAAAGGATCTTCTTGAGATCCTTTTTTTCTGCGCGTAATCTGCTGCTTGCAAACAAAAAAACCAC
CGCTACCAGCGGTGGTTTGTTTGCCGGATCAAGAGCTACCAACTCTTTTTCCGAAGGTAACTGGCTTCAGCAGAGCGCAGATACCAAATACTGTCCT
TCTAGTGTAGCCGTAGTTAGGCCACCACTTCAAGAACTCTGTAGCACCGCCTACATACCTCGCTCTGCTAATCCTGTTACCAGTGGCTGCTGCCAGT
GGCGATAAGTCGTGTCTTACCGGGTTGGACTCAAGACGATAGTTACCGGATAAGGCGCAGCGGTCGGGCTGAACGGGGGGTTCGTGCACACAGCCCA
GCTTGGAGCGAACGACCTACACCGAACTGAGATACCTACAGCGTGAGCTATGAGAAAGCGCCACGCTTCCCGAAGGGAGAAAGGCGGACAGGTATCC
GGTAAGCGGCAGGGTCGGAACAGGAGAGCGCACGAGGGAGCTTCCAGGGGGAAACGCCTGGTATCTTTATAGTCCTGTCGGGTTTCGCCACCTCTGA
CTTGAGCGTCGATTTTTGTGATGCTCGTCAGGGGGGCGGAGCCTATGGAAAAACGCCAGCAACGCGGCCTTTTTACGGTTCCTGGCCTTTTGCTGGC
CTTTTGCTCACATGTTCTTTCCTGCGTTATCCCCTGATTCTGTGGATAACCGTATTACCGCCTTTGAGTGAGCTGATACCGCTCGCCGCAGCCGAAC
GACCGAGCGCAGCGAGTCAGTGAGCGAGGAAGCGGAAGAGCGCCTGATGCGGTATTTTCTCCTTACGCATCTGTGCGGTATTTCACACCGCATATGG
TGCACTCTCAGTACAATCTGCTCTGATGCCGCATAGTTAAGCCAGTATACACTCCGCTATCGCTACGTGACTGGGTCATGGCTGCGCCCCGACACCC
GCCAACACCCGCTGACGCGCCCTGACGGGCTTGTCTGCTCCCGGCATCCGCTTACAGACAAGCTGTGACCGTCTCCGGGAGCTGCATGTGTCAGAGG
TTTTCACCGTCATCACCGAAACGCGCGAGGCAGGGTGCCTTGATGTGGGCGCCGGCGGTCGAGTGGCGACGGCGCGGCTTGTCCGCGCCCTGGTAGA
TTGCCTGGCCGTAGGCCAGCCATTTTTGAGCGGCCAGCGGCCGCGATAGGCCGACGCGAAGCGGCGGGGCGTAGGGAGCGCAGCGACCGAAGGGTAG
GCGCTTTTTGCAGCTCTTCGGCTGTGCGCTGGCCAGACAGTTATGCACAGGCCAGGCGGGTTTTAAGAGTTTTAATAAGTTTTAAAGAGTTTTAGGC
GGAAAAATCGCCTTTTTTCTCTTTTATATCAGTCACTTACATGTGTGACCGGTTCCCAATGTACGGCTTTGGGTTCCCAATGTACGGGTTCCGGTTC
CCAATGTACGGCTTTGGGTTCCCAATGTACGTGCTATCCACAGGAAACAGACCTTTTCGACCTTTTTCCCCTGCTAGGGCAATTTGCCCTAGCATCT
GCTCCGTACATTAGGAACCGGCGGATGCTTCGCCCTCGATCAGGTTGCGGTAGCGCATGACTAGGATCGGGCCAGCCTGCCCCGCCTCCTCCTTCAA
ATCGTACTCCGGCAGGTCATTTGACCCGATCAGCTTGCGCACGGTGAAACAGAACTTCTTGAACTCTCCGGCGCTGCCACTGCGTTCGTAGATCGTC
TTGAACAACCATCTGGCTTCTGCCTTGCCTGCGGCGCGGCGTGCCAGGCGGTAGAGAAAACGGCCGATGCCGGGATCGATCAAAAAGTAATCGGGGT
GAACCGTCAGCACGTCCGGGTTCTTGCCTTCTGTGATCTCGCGGTACATCCAATCAGCTAGCTCGATCTCGATGTACTCCGGCCGCCCGGTTTCGCT
CTTTACGATCTTGTAGCGGCTAATCAAGGCTTCACCCTCGGATACCGTCACCAGGCGGCCGTTCTTGGCCTTCTTCGTACGCTGCATGGCAACGTGC
GTGGTGTTTAACCGAATGCAGGTTTCTACCAGGTCGTCTTTCTGCTTTCCGCCATCGGCTCGCCGGCAGAACTTGAGTACGTCCGCAACGTGTGGAC
GGAACACGCGGCCGGGCTTGTCTCCCTTCCCTTCCCGGTATCGGTTCATGGATTCGGTTAGATGGGAAACCGCCATCAGTACCAGGTCGTAATCCCA
CACACTGGCCATGCCGGCCGGCCCTGCGGAAACCTCTACGTGCCCGTCTGGAAGCTCGTAGCGGATCACCTCGCCAGCTCGTCGGTCACGCTTCGAC
AGACGGAAAACGGCCACGTCCATGATGCTGCGACTATCGCGGGTGCCCACGTCATAGAGCATCGGAACGAAAAAATCTGGTTGCTCGTCGCCCTTGG
GCGGCTTCCTAATCGACGGCGCACCGGCTGCCGGCGGTTGCCGGGATTCTTTGCGGATTCGATCAGCGGCCGCTTGCCACGATTCACCGGGGCGTGC
TTCTGCCTCGATGCGTTGCCGCTGGGCGGCCTGCGCGGCCTTCAACTTCTCCACCAGGTCATCACCCAGCGCCGCGCCGATTTGTACCGGGCCGGAT
GGTTTGCGACCGTCACGCCGATTCCTCGGGCTTGGGGGTTCCAGTGCCATTGCAGGGCCGGCAGACAACCCAGCCGCTTACGCCTGGCCAACCGCCC
GTTCCTCCACACATGGGGCATTCCACGGCGTCGGTGCCTGGTTGTTCTTGATTTTCCATGCCGCCTCCTTTAGCCGCTAAAATTCATCTACTCATTT
ATTCATTTGCTCATTTACTCTGGTAGCTGCGCGATGTATTCAGATAGCAGCTCGGTAATGGTCTTGCCTTGGCGTACCGCGTACATCTTCAGCTTGG
TGTGATCCTCCGCCGGCAACTGAAAGTTGACCCGCTTCATGGCTGGCGTGTCTGCCAGGCTGGCCAACGTTGCAGCCTTGCTGCTGCGTGCGCTCGG
ACGGCCGGCACTTAGCGTGTTTGTGCTTTTGCTCATTTTCTCTTTACCTCATTAACTCAAATGAGTTTTGATTTAATTTCAGCGGCCAGCGCCTGGA
CCTCGCGGGCAGCGTCGCCCTCGGGTTCTGATTCAAGAACGGTTGTGCCGGCGGCGGCAGTGCCTGGGTAGCTCACGCGCTGCGTGATACGGGACTC
AAGAATGGGCAGCTCGTACCCGGCCAGCGCCTCGGCAACCTCACCGCCGATGCGCGTGCCTTTGATCGCCCGCGACACGACAAAGGCCGCTTGTAGC
CTTCCATCCGTGACCTCAATGCGCTGCTTAACCAGCTCCACCAGGTCGGCGGTGGCCCATATGTCGTAAGGGCTTGGCTGCACCGGAATCAGCACGA
AGTCGGCTGCCTTGATCGCGGACACAGCCAAGTCCGCCGCCTGGGGCGCTCCGTCGATCACTACGAAGTCGCGCCGGCCGATGGCCTTCACGTCGCG
GTCAATCGTCGGGCGGTCGATGCCGACAACGGTTAGCGGTTGATCTTCCCGCACGGCCGCCCAATCGCGGGCACTGCCCTGGGGATCGGAATCGACT
AACAGAACATCGGCCCCGGCGAGTTGCAGGGCGCGGGCTAGATGGGTTGCGATGGTCGTCTTGCCTGACCCGCCTTTCTGGTTAAGTACAGCGATAA
CCTTCATGCGTTCCCCTTGCGTATTTGTTTATTTACTCATCGCATCATATACGCAGCGACCGCATGACGCAAGCTGTTTTACTCAAATACACATCAC
CTTTTTAGACGGCGGCGCTCGGTTTCTTCAGCGGCCAAGCTGGCCGGCCAGGCCGCCAGCTTGGCATCAGACAAACCGGCCAGGATTTCATGCAGCC
GCACGGTTGAGACGTGCGCGGGCGGCTCGAACACGTACCCGGCCGCGATCATCTCCGCCTCGATCTCTTCGGTAATGAAAAACGGTTCGTCCTGGCC
GTCCTGGTGCGGTTTCATGCTTGTTCCTCTTGGCGTTCATTCTCGGCGGCCGCCAGGGCGTCGGCCTCGGTCAATGCGTCCTCACGGAAGGCACCGC
GCCGCCTGGCCTCGGTGGGCGTCACTTCCTCGCTGCGCTCAAGTGCGCGGTACAGGGTCGAGCGATGCACGCCAAGCAGTGCAGCCGCCTCTTTCAC
GGTGCGGCCTTCCTGGTCGATCAGCTCGCGGGCGTGCGCGATCTGTGCCGGGGTGAGGGTAGGGCGGGGGCCAAACTTCACGCCTCGGGCCTTGGCG
GCCTCGCGCCCGCTCCGGGTGCGGTCGATGATTAGGGAACGCTCGAACTCGGCAATGCCGGCGAACACGGTCAACACCATGCGGCCGGCCGGCGTGG
TGGTGTCGGCCCACGGCTCTGCCAGGCTACGCAGGCCCGCGCCGGCCTCCTGGATGCGCTCGGCAATGTCCAGTAGGTCGCGGGTGCTGCGGGCCAG
GCGGTCTAGCCTGGTCACTGTCACAACGTCGCCAGGGCGTAGGTGGTCAAGCATCCTGGCCAGCTCCGGGCGGTCGCGCCTGGTGCCGGTGATCTTC
TCGGAAAACAGCTTGGTGCAGCCGGCCGCGTGCAGTTCGGCCCGTTGGTTGGTCAAGTCCTGGTCGTCGGTGCTGACGCGGGCATAGCCCAGCAGGC
CAGCGGCGGCGCTCTTGTTCATGGCGTAATGTCTCCGGTTCTAGTCGCAAGTATTCTACTTTATGCGACTAAAACACGCGACAAGAAAACGCCAGGA
AAAGGGCAGGGCGGCAGCCTGTCGCGTAACTTAGGACTTGTGCGACATGTCGTTTTCAGAAGACGGCTGCACTGAACGTCAGAAGCCGACTGCACTA
TAGCAGCGGAGGGGTTGGATCAAAGTACTTTGATCCCGAGGGGAACCCTGTGGTTGGCATGCACATACAAATGGACGAACGGATAAACCTTTTCACG

Supplementary Sequence S1. DNA sequence of the all-in-one expressing vector pLR3850 used for making RUBY-ABEs (continued 1).

CCCTTTTAAATATCCGTTATTCTAATAAACGCTCTTTTCTCTTAGGTTTACCCGCCAATATATCCTGTCAAACACTGATAGTTTAAACTGAAGGCGG
GAAACGACAATCTGATCCAAGCTCAAGCTGCTCTAGCATTCGCCATTCAGGCTGCGCAACTGTTGGGAAGGGCGATCGGTGCGGGCCTCTTCGCTAT
TACGCCAGCTGGCGAAAGGGGGATGTGCTGCAAGGCGATTAAGTTGGGTAACGCCAGGGTTTTCCCAGTCACGACGTTGTAAAACGACGGCCAGTGC
CAAGCTTGGCGTGCCTGCAGGTCAACATGGTGGAGCACGACACACTTGTCTACTCCAAAAATATCAAAGATACAGTCTCAGAAGACCAAAGGGCAAT
TGAGACTTTTCAACAAAGGGTAATATCCGGAAACCTCCTCGGATTCCATTGCCCAGCTATCTGTCACTTTATTGTGAAGATAGTGGAAAAGGAAGGT
GGCTCCTACAAATGCCATCATTGCGATAAAGGAAAGGCCATCGTTGAAGATGCCTCTGCCGACAGTGGTCCCAAAGATGGACCCCCACCCACGAGGA
GCATCGTGGAAAAAGAAGACGTTCCAACCACGTCTTCAAAGCAAGTGGATTGATGTGATAACATGGTGGAGCACGACACACTTGTCTACTCCAAAAA
TATCAAAGATACAGTCTCAGAAGACCAAAGGGCAATTGAGACTTTTCAACAAAGGGTAATATCCGGAAACCTCCTCGGATTCCATTGCCCAGCTATC
TGTCACTTTATTGTGAAGATAGTGGAAAAGGAAGGTGGCTCCTACAAATGCCATCATTGCGATAAAGGAAAGGCCATCGTTGAAGATGCCTCTGCCG
ACAGTGGTCCCAAAGATGGACCCCCACCCACGAGGAGCATCGTGGAAAAAGAAGACGTTCCAACCACGTCTTCAAAGCAAGTGGATTGATGTGATAT
CTCCACTGACGTAAGGGATGACGCACAATCCCACTATCCTTCGCAAGACCCTTCCTCTATATAAGGAAGTTCATTTCATTTGGAGAGGACCTCGACT
CTAGAGGATCCCCGGGTACCGGGCCCCCCCTCGAGGCGCGCCAAGCTATCAAACAAGTTTGTACAAAAAAGCAGGCTCCGATGAAACGGACAGCCGA
CGGAAGCGAGTTCGAGTCACCAAAGAAGAAGCGGAAAGTCTCTGAGGTGGAGTTTTCCCACGAGTACTGGATGAGACATGCCCTGACCCTGGCCAAG
AGGGCACGGGATGAGAGGGAGGTGCCTGTGGGAGCCGTGCTGGTGCTGAACAATAGAGTGATCGGCGAGGGCTGGAACAGAGCCATCGGCCTGCACG
ACCCAACAGCCCATGCCGAAATTATGGCCCTGAGACAGGGCGGCCTGGTCATGCAGAACTACAGACTGATTGACGCCACCCTGTACGTGACATTCGA
GCCTTGCGTGATGTGCGCCGGCGCCATGATCCACTCTAGGATCGGCCGCGTGGTGTTTGGCGTGAGGAACTCAAAAAGAGGCGCCGCAGGCTCCCTG
ATGAACGTGCTGAACTACCCCGGCATGAATCACCGCGTCGAAATTACCGAGGGAATCCTGGCAGATGAATGTGCCGCCCTGCTGTGCGATTTCTATC
GGATGCCTAGACAGGTGTTCAATGCTCAGAAGAAGGCCCAGAGCTCCATCAACTCCGGAGGATCTAGCGGAGGCTCCTCTGGCTCTGAGACACCTGG
CACAAGCGAGAGCGCAACACCTGAAAGCAGCGGGGGCAGCAGCGGGGGGTCAACCATGGATTACAAGGACCACGACGGGGATTACAAGGACCACGAC
ATTGATTACAAGGATGATGATGACAAGATGGCTCCGAAGAAGAAGAGGAAGGTTGGCATCCACGGGGTGCCAGCTGCTGACAAGAAGTACTCGATCG
GCCTCGCTATTGGGACTAACTCTGTTGGCTGGGCCGTGATCACCGACGAGTACAAGGTGCCCTCAAAGAAGTTCAAGGTCCTGGGCAACACCGATCG
GCATTCCATCAAGAAGAATCTCATTGGCGCTCTCCTGTTCGACAGCGGCGAGACGGCTGAGGCTACGCGGCTCAAGCGCACCGCCCGCAGGCGGTAC
ACGCGCAGGAAGAATCGCATCTGCTACCTGCAGGAGATTTTCTCCAACGAGATGGCGAAGGTTGACGATTCTTTCTTCCACAGGCTGGAGGAGTCAT
TCCTCGTGGAGGAGGATAAGAAGCACGAGCGGCATCCAATCTTCGGCAACATTGTCGACGAGGTTGCCTACCACGAGAAGTACCCTACGATCTACCA
TCTGCGGAAGAAGCTCGTGGACTCCACAGATAAGGCGGACCTCCGCCTGATCTACCTCGCTCTGGCCCACATGATTAAGTTCAGGGGCCATTTCCTG
ATCGAGGGGGATCTCAACCCGGACAATAGCGATGTTGACAAGCTGTTCATCCAGCTCGTGCAGACGTACAACCAGCTCTTCGAGGAGAACCCCATTA
ATGCGTCAGGCGTCGACGCGAAGGCTATCCTGTCCGCTAGGCTCTCGAAGTCTCGGCGCCTCGAGAACCTGATCGCCCAGCTGCCGGGCGAGAAGAA
GAACGGCCTGTTCGGGAATCTCATTGCGCTCAGCCTGGGGCTCACGCCCAACTTCAAGTCGAATTTCGATCTCGCTGAGGACGCCAAGCTGCAGCTC
TCCAAGGACACATACGACGATGACCTGGATAACCTCCTGGCCCAGATCGGCGATCAGTACGCGGACCTGTTCCTCGCTGCCAAGAATCTGTCGGACG
CCATCCTCCTGTCTGATATTCTCAGGGTGAACACCGAGATTACGAAGGCTCCGCTCTCAGCCTCCATGATCAAGCGCTACGACGAGCACCATCAGGA
TCTGACCCTCCTGAAGGCGCTGGTCAGGCAGCAGCTCCCCGAGAAGTACAAGGAGATCTTCTTCGATCAGTCGAAGAACGGCTACGCTGGGTACATT
GACGGCGGGGCCTCTCAGGAGGAGTTCTACAAGTTCATCAAGCCGATTCTGGAGAAGATGGACGGCACGGAGGAGCTGCTGGTGAAGCTCAATCGCG
AGGACCTCCTGAGGAAGCAGCGGACATTCGATAACGGCAGCATCCCACACCAGATTCATCTCGGGGAGCTGCACGCTATCCTGAGGAGGCAGGAGGA
CTTCTACCCTTTCCTCAAGGATAACCGCGAGAAGATCGAGAAGATTCTGACTTTCAGGATCCCGTACTACGTCGGCCCACTCGCTAGGGGCAACTCC
CGCTTCGCTTGGATGACCCGCAAGTCAGAGGAGACGATCACGCCGTGGAACTTCGAGGAGGTGGTCGACAAGGGCGCTAGCGCTCAGTCGTTCATCG
AGAGGATGACGAATTTCGACAAGAACCTGCCAAATGAGAAGGTGCTCCCTAAGCACTCGCTCCTGTACGAGTACTTCACAGTCTACAACGAGCTGAC
TAAGGTGAAGTATGTGACCGAGGGCATGAGGAAGCCGGCTTTCCTGTCTGGGGAGCAGAAGAAGGCCATCGTGGACCTCCTGTTCAAGACCAACCGG
AAGGTCACGGTTAAGCAGCTCAAGGAGGACTACTTCAAGAAGATTGAGTGCTTCGATTCGGTCGAGATCTCTGGCGTTGAGGACCGCTTCAACGCCT
CCCTGGGGACCTACCACGATCTCCTGAAGATCATTAAGGATAAGGACTTCCTGGACAACGAGGAGAATGAGGATATCCTCGAGGACATTGTGCTGAC
ACTCACTCTGTTCGAGGACCGGGAGATGATCGAGGAGCGCCTGAAGACTTACGCCCATCTCTTCGATGACAAGGTCATGAAGCAGCTCAAGAGGAGG
AGGTACACCGGCTGGGGGAGGCTGAGCAGGAAGCTCATCAACGGCATTCGGGACAAGCAGTCCGGGAAGACGATCCTCGACTTCCTGAAGAGCGATG
GCTTCGCGAACCGCAATTTCATGCAGCTGATTCACGATGACAGCCTCACATTCAAGGAGGATATCCAGAAGGCTCAGGTGAGCGGCCAGGGGGACTC
GCTGCACGAGCATATCGCGAACCTCGCTGGCTCGCCAGCTATCAAGAAGGGGATTCTGCAGACCGTGAAGGTTGTGGACGAGCTGGTGAAGGTCATG
GGCAGGCACAAGCCTGAGAACATCGTCATTGAGATGGCCCGGGAGAATCAGACCACGCAGAAGGGCCAGAAGAACTCACGCGAGAGGATGAAGAGGA
TCGAGGAGGGCATTAAGGAGCTGGGGTCCCAGATCCTCAAGGAGCACCCGGTGGAGAACACGCAGCTGCAGAATGAGAAGCTCTACCTGTACTACCT
CCAGAATGGCCGCGATATGTATGTGGACCAGGAGCTGGATATTAACAGGCTCAGCGATTACGACGTCGATCATATCGTTCCACAGTCATTCCTGAAG
GATGACTCCATTGACAACAAGGTCCTCACCAGGTCGGACAAGAACCGGGGCAAGTCTGATAATGTTCCTTCAGAGGAGGTCGTTAAGAAGATGAAGA
ACTACTGGCGCCAGCTCCTGAATGCCAAGCTGATCACGCAGCGGAAGTTCGATAACCTCACAAAGGCTGAGAGGGGCGGGCTCTCTGAGCTGGACAA
GGCGGGCTTCATCAAGAGGCAGCTGGTCGAGACACGGCAGATCACTAAGCACGTTGCGCAGATTCTCGACTCACGGATGAACACTAAGTACGATGAG
AATGACAAGCTGATCCGCGAGGTGAAGGTCATCACCCTGAAGTCAAAGCTCGTCTCCGACTTCAGGAAGGATTTCCAGTTCTACAAGGTTCGGGAGA
TCAACAATTACCACCATGCCCATGACGCGTACCTGAACGCGGTGGTCGGCACAGCTCTGATCAAGAAGTACCCAAAGCTCGAGAGCGAGTTCGTGTA
CGGGGACTACAAGGTTTACGATGTGAGGAAGATGATCGCCAAGTCGGAGCAGGAGATTGGCAAGGCTACCGCCAAGTACTTCTTCTACTCTAACATT
ATGAATTTCTTCAAGACAGAGATCACTCTGGCCAATGGCGAGATCCGGAAGCGCCCCCTCATCGAGACGAACGGCGAGACGGGGGAGATCGTGTGGG
ACAAGGGCAGGGATTTCGCGACCGTCAGGAAGGTTCTCTCCATGCCACAAGTGAATATCGTCAAGAAGACAGAGGTCCAGACTGGCGGGTTCTCTAA
GGAGTCAATTCTGCCTAAGCGGAACAGCGACAAGCTCATCGCCCGCAAGAAGGACTGGGATCCGAAGAAGTACGGCGGGTTCGACAGCCCCACTGTG
GCCTACTCGGTCCTGGTTGTGGCGAAGGTTGAGAAGGGCAAGTCCAAGAAGCTCAAGAGCGTGAAGGAGCTGCTGGGGATCACGATTATGGAGCGCT
CCAGCTTCGAGAAGAACCCGATCGATTTCCTGGAGGCGAAGGGCTACAAGGAGGTGAAGAAGGACCTGATCATTAAGCTCCCCAAGTACTCACTCTT
CGAGCTGGAGAACGGCAGGAAGCGGATGCTGGCTTCCGCTGGCGAGCTGCAGAAGGGGAACGAGCTGGCTCTGCCGTCCAAGTATGTGAACTTCCTC
TACCTGGCCTCCCACTACGAGAAGCTCAAGGGCAGCCCCGAGGACAACGAGCAGAAGCAGCTGTTCGTCGAGCAGCACAAGCATTACCTCGACGAGA
TCATTGAGCAGATTTCCGAGTTCTCCAAGCGCGTGATCCTGGCCGACGCGAATCTGGATAAGGTCCTCTCCGCGTACAACAAGCACCGCGACAAGCC
AATCAGGGAGCAGGCTGAGAATATCATTCATCTCTTCACCCTGACGAACCTCGGCGCCCCTGCTGCTTTCAAGTACTTCGACACAACTATCGATCGC
AAGAGGTACACAAGCACTAAGGAGGTCCTGGACGCGACCCTCATCCACCAGTCGATTACCGGCCTCTACGAGACGCGCATCGACCTGTCTCAGCTCG
GGGGCGACAAGCGGCCAGCGGCGACGAAGAAGGCGGGGCAGGCGAAGAAGAAGAAGTGAGCTCAGAGCTTTCGTTCGTATCATCGGTTTCGACAACG
TTCGTCAAGTTCAATGCATCAGTTTCATTGCGCACACACCAGAATCCTACTGAGTTTGAGTATTATGGCATTGGGAAAACTGTTTTTCTTGTACCAT

Supplementary Sequence S1. DNA sequence of the all-in-one expressing vector pLR3850 used for making RUBY-ABEs (continued 2).

TTGTTGTGCTTGTAATTTACTGTGTTTTTTATTCGGTTTTCGCTATCGAACTGTGAAATGGAAATGGATGGAGAAGAGTTAATGAATGATATGGTCC
TTTTGTTCATTCTCAAATTAATATTATTTGTTTTTTCTCTTATTTGTTGTGTGTTGAATTTGAAATTATAAGAGATATGCAAACATTTTGTTTTGAG
TAAAAATGTGTCAAATCGTGGCCTCTAATGACCGAAGTTAATATGAGGAGTAAAACACTTGTAGTTGTACCATTATGCTTATTCACTAGGCAACAAA
TATATTTTCAGACCTAGAAAAGCTGCAAATGTTACTGAATACAAGTATGTCCTCTTGTGTTTTAGACATTTATGAACTTTCCTTTATGTAATTTTCC
AGAATCCTTGTCAGATTCTAATCATTGCTTTATAATTATAGTTATACTCATGGATTTGTAGTTGAGTATGAAAATATTTTTTAATGCATTTTATGAC
TTGCCAATTGTTCCGGAACTCTAGATAAGCTTACCGGAAAGGGCGAATTCGCAACTTTGTATACAAAAGTTGCCCCATGGCGTTCCCTCTAGATAAC
GCAGGATCCTATTTACTTTAAATTTTTCTTATGGCTCAGCCTGTGATGGATAACTGAATCAAACAAATGGCGTCTGGGTTTAAGAACATCTGTTTTG
GCTATGTTGGACGAAACAAGTGAACTTTTAGGATCAACTTCCGTTTATATACGGAGCTTATATCGAGCAATAAGATAAGTGGGCTTTTTATGTAATT
TAATGGGCTATCGTCCATATATTCACTAATACCCATGCCCAGTACCCATGTATGCGTTTCATATAAGCTCCTAATTTCTCCCACATCGCTCAAATCT
AAACAAATCTTGTTGTATATATAACACTGAGGGAGCACCATTGGTCAGAGACCGGCGCCGCTACAGGGCGCGTCCCATTCGCCATTCAGGCTGCGCA
ACTGTTGGGAAGGGCGATCGGTGCGGGCCTCTTCGCTATTACGCCAGCTGGCGAAAGGGGGATGTGCTGCAAGGCGATTAAGTTGGGTAACGCCAGG
GTTTTCCCAGTCACGACGTTGTAAAACGACGGCCAGTGAGCGCGCGTAATACGACTCACTATAGGGCGAATTGGGTACCGGGCCCCCCCTCGAGGTC
CTCCAGCTTTTGTTCCCTTTAGTGAGGGTTAATTGCGCGCTTGGCGTAATCATGGTCATAGCTGTTTCCTGTGTGAAATTGTTATCCGCTCACAATT
CCACACAACATACGAGCCGGAAGCATAAAGTGTAAAGCCTGGGGTGCCTAATGAGTGAGCTAACTCACATTAATTGCGTTGCGCTCACTGCCCGCTT
TCCACCGGTGGTCTCTGTTTTAGAGCTAGAAATAGCAAGTTAAAATAAGGCTAGTCCGTTATCAACTTGAAAAAGTGGCACCGAGTCGGTGCTTTTT
TTTCCCTTTCCTTTTTTCTTTTTTTTGCCATAAACTTAAATTTGTATATCGATCATTGTAGATATTGAAAACCTAGAACAAACCAACATCCATGTGA
ATGTCTTTCATGACTGATTTAGAGATAATTCTTGAATTTTGGAACTAGAATCTATAATGAGCCTAAATTAAAACATTGTGAACTAGTAAGGGCGAAT
TCGACCCAGCTTTCTTGTACAAAGTGGTTCGATAATTCTTAATTAACTAGTTCTAGAGCGGCCGCCACCGCGGTGGAGCTCGAATTTCCCCGATCGT
TCAAACATTTGGCAATAAAGTTTCTTAAGATTGAATCCTGTTGCCGGTCTTGCGATGATTATCATATAATTTCTGTTGAATTACGTTAAGCATGTAA
TAATTAACATGTAATGCATGACGTTATTTATGAGATGGGTTTTTATGATTAGAGTCCCGCAATTATACATTTAATACGCGATAGAAAACAAAATATA
GCGCGCAAACTAGGATAAATTATCGCGCGCGGTGTCATCTATGTTACTGAATTCGTAATCATGGTCATAGAAGCTTGCATGCCTGCAGGTCGACTCT
AGAGGATCCCCGGGTACCGAGCTCGAATTCGTAATCATGTCATAGCTGTTTCCTGTGTGAAATTGTTATCCGCTCACAATTCCACACAACATACGAG
CCGGAAGCATAAAGTGTAAAGCCTGGGGTGCCTAATGAGTGAGCTAACTCACATTAATTGCGTTGCGCTCACTGCCCGCTTTCCAGTCGGGAAACCT
GTCGTGCCAGCTGCATTAATGAATCGGCCAACGCGCGGGGAGAGGCGGTTTGCGTATTGGCTAGAGCAGCTTGCCAACATGGTGGAGCACGACACTC
TCGTCTACTCCAAGAATATCAAAGATACAGTCTCAGAAGACCAAAGGGCTATTGAGACTTTTCAACAAAGGGTAATATCGGGAAACCTCCTCGGATT
CCATTGCCCAGCTATCTGTCACTTCATCAAAAGGACAGTAGAAAAGGAAGGTGGCACCTACAAATGCCATCATTGCGATAAAGGAAAGGCTATCGTT
CAAGATGCCTCTGCCGACAGTGGTCCCAAAGATGGACCCCCACCCACGAGGAGCATCGTGGAAAAAGAAGACGTTCCAACCACGTCTTCAAAGCAAG
TGGATTGATGTGATAACATGGTGGAGCACGACACTCTCGTCTACTCCAAGAATATCAAAGATACAGTCTCAGAAGACCAAAGGGCTATTGAGACTTT
TCAACAAAGGGTAATATCGGGAAACCTCCTCGGATTCCATTGCCCAGCTATCTGTCACTTCATCAAAAGGACAGTAGAAAAGGAAGGTGGCACCTAC
AAATGCCATCATTGCGATAAAGGAAAGGCTATCGTTCAAGATGCCTCTGCCGACAGTGGTCCCAAAGATGGACCCCCACCCACGAGGAGCATCGTGG
AAAAAGAAGACGTTCCAACCACGTCTTCAAAGCAAGTGGATTGATGTGATATCTCCACTGACGTAAGGGATGACGCACAATCCCACTATCCTTCGCA
AGACCTTCCTCTATATAAGGAAGTTCATTTCATTTGGAGAGGACACGCTGAAATCACCAGTCTCTCTCTACAAATCTATCTCTCTCGAGCTTTCGCA
GATCTGTCGATCGACCATGGGGATTGAACAAGATGGATTGCACGCAGGTTCTCCGGCCGCTTGGGTGGAGAGGCTATTCGGCTATGACTGGGCACAA
CAGACAATCGGCTGCTCTGATGCCGCCGTGTTCCGGCTGTCAGCGCAGGGGCGCCCGGTTCTTTTTGTCAAGACCGACCTGTCCGGTGCCCTGAATG
AACTCCAGGACGAGGCAGCGCGGCTATCGTGGCTGGCCACGACGGGCGTTCCTTGCGCAGCTGTGCTCGACGTTGTCACTGAAGCGGGAAGGGACTG
GCTGCTATTGGGCGAAGTGCCGGGGCAGGATCTCCTGTCATCTCACCTTGCTCCTGCCGAGAAAGTATCCATCATGGCTGATGCAATGCGGCGGCTG
CATACGCTTGATCCGGCTACCTGCCCATTCGACCACCAAGCGAAACATCGCATCGAGCGAGCACGTACTCGGATGGAAGCCGGTCTTGTCGATCAGG
ATGATCTGGACGAAGAGCATCAGGGGCTCGCGCCAGCCGAACTGTTCGCCAGGCTCAAGGCGCGCATGCCCGACGGCGAGGATCTCGTCGTGACACA
TGGCGATGCCTGCTTGCCGAATATCATGGTGGAAAATGGCCGCTTTTCTGGATTCATCGACTGTGGCCGGCTGGGTGTGGCGGACCGCTATCAGGAC
ATAGCGTTGGCTACCCGTGATATTGCTGAAGAGCTTGGCGGCGAATGGGCTGACCGCTTCCTCGTGCTTTACGGTATCGCCGCTCCCGATTCGCAGC
GCATCGCCTTCTATCGCCTTCTTGACGAGTTCTTCTGAGCGGGACTCTGGGGTTCGGATCGATCCTCTAGCTAGAGTCGATCGACAAGCTCGAGTTT
CTCCATAATAATGTGTGAGTAGTTCCCAGATAAGGGAATTAGGGTTCCTATAGGGTTTCGCTCATGTGTTGAGCATATAAGAAACCCTTAGTATGTA
TTTGTATTTGTAAAATACTTCTATCAATAAAATTTCTAATTCCTAAAACCAAAATCCAGTACTAAAATCCAGATCCCCCGAATTAATTCGGCGTTAA
TTCAGTACATTAAAAACGTCCGCAATGTGTTATTAAGTTGTCTAAGCGTCAATTTGTTTACACCACAATATATCCTGCCA

Supplementary Sequence S2. The DNA sequence of the binary vector pCGS710 provided by Drs. Daniel Voytas and Colby Starker.

CCAGCCAGCCAACAGCTCCCCGACCGGCAGCTCGGCACAAAATCACCACTCGATACAGGCAGCCCATCAGTCCGGGACGGCGTCAGCGGGAGAGCCG
TTGTAAGGCGGCAGACTTTGCTCATGTTACCGATGCTATTCGGAAGAACGGCAACTAAGCTGCCGGGTTTGAAACACGGATGATCTCGCGGAGGGTA
GCATGTTGATTGTAACGATGACAGAGCGTTGCTGCCTGTGATCACCGCGGTTTCAAAATCGGCTCCGTCGATACTATGTTATACGCCAACTTTGAAA
ACAACTTTGAAAAAGCTGTTTTCTGGTATTTAAGGTTTTAGAATGCAAGGAACAGTGAATTGGAGTTCGTCTTGTTATAATTAGCTTCTTGGGGTAT
CTTTAAATACTGTAGAAAAGAGGAAGGAAATAATAAATGGCTAAAATGAGAATATCACCGGAATTGAAAAAACTGATCGAAAAATACCGCTGCGTAA
AAGATACGGAAGGAATGTCTCCTGCTAAGGTATATAAGCTGGTGGGAGAAAATGAAAACCTATATTTAAAAATGACGGACAGCCGGTATAAAGGGAC
CACCTATGATGTGGAACGGGAAAAGGACATGATGCTATGGCTGGAAGGAAAGCTGCCTGTTCCAAAGGTCCTGCACTTTGAACGGCATGATGGCTGG
AGCAATCTGCTCATGAGTGAGGCCGATGGCGTCCTTTGCTCGGAAGAGTATGAAGATGAACAAAGCCCTGAAAAGATTATCGAGCTGTATGCGGAGT
GCATCAGGCTCTTTCACTCCATCGACATATCGGATTGTCCCTATACGAATAGCTTAGACAGCCGCTTAGCCGAATTGGATTACTTACTGAATAACGA
TCTGGCCGATGTGGATTGCGAAAACTGGGAAGAAGACACTCCATTTAAAGATCCGCGCGAGCTGTATGATTTTTTAAAGACGGAAAAGCCCGAAGAG
GAACTTGTCTTTTCCCACGGCGACCTGGGAGACAGCAACATCTTTGTGAAAGATGGCAAAGTAAGTGGCTTTATTGATCTTGGGAGAAGCGGCAGGG
CGGACAAGTGGTATGACATTGCCTTCTGCGTCCGGTCGATCAGGGAGGATATCGGGGAAGAACAGTATGTCGAGCTATTTTTTGACTTACTGGGGAT
CAAGCCTGATTGGGAGAAAATAAAATATTATATTTTACTGGATGAATTGTTTTAGTACCTAGAATGCATGACCAAAATCCCTTAACGTGAGTTTTCG
TTCCACTGAGCGTCAGACCCCGTAGAAAAGATCAAAGGATCTTCTTGAGATCCTTTTTTTCTGCGCGTAATCTGCTGCTTGCAAACAAAAAAACCAC
CGCTACCAGCGGTGGTTTGTTTGCCGGATCAAGAGCTACCAACTCTTTTTCCGAAGGTAACTGGCTTCAGCAGAGCGCAGATACCAAATACTGTCCT
TCTAGTGTAGCCGTAGTTAGGCCACCACTTCAAGAACTCTGTAGCACCGCCTACATACCTCGCTCTGCTAATCCTGTTACCAGTGGCTGCTGCCAGT
GGCGATAAGTCGTGTCTTACCGGGTTGGACTCAAGACGATAGTTACCGGATAAGGCGCAGCGGTCGGGCTGAACGGGGGGTTCGTGCACACAGCCCA
GCTTGGAGCGAACGACCTACACCGAACTGAGATACCTACAGCGTGAGCTATGAGAAAGCGCCACGCTTCCCGAAGGGAGAAAGGCGGACAGGTATCC
GGTAAGCGGCAGGGTCGGAACAGGAGAGCGCACGAGGGAGCTTCCAGGGGGAAACGCCTGGTATCTTTATAGTCCTGTCGGGTTTCGCCACCTCTGA
CTTGAGCGTCGATTTTTGTGATGCTCGTCAGGGGGGCGGAGCCTATGGAAAAACGCCAGCAACGCGGCCTTTTTACGGTTCCTGGCCTTTTGCTGGC
CTTTTGCTCACATGTTCTTTCCTGCGTTATCCCCTGATTCTGTGGATAACCGTATTACCGCCTTTGAGTGAGCTGATACCGCTCGCCGCAGCCGAAC
GACCGAGCGCAGCGAGTCAGTGAGCGAGGAAGCGGAAGAGCGCCTGATGCGGTATTTTCTCCTTACGCATCTGTGCGGTATTTCACACCGCATATGG
TGCACTCTCAGTACAATCTGCTCTGATGCCGCATAGTTAAGCCAGTATACACTCCGCTATCGCTACGTGACTGGGTCATGGCTGCGCCCCGACACCC
GCCAACACCCGCTGACGCGCCCTGACGGGCTTGTCTGCTCCCGGCATCCGCTTACAGACAAGCTGTGACCGTCTCCGGGAGCTGCATGTGTCAGAGG
TTTTCACCGTCATCACCGAAACGCGCGAGGCAGGGTGCCTTGATGTGGGCGCCGGCGGTCGAGTGGCGACGGCGCGGCTTGTCCGCGCCCTGGTAGA
TTGCCTGGCCGTAGGCCAGCCATTTTTGAGCGGCCAGCGGCCGCGATAGGCCGACGCGAAGCGGCGGGGCGTAGGGAGCGCAGCGACCGAAGGGTAG
GCGCTTTTTGCAGCTCTTCGGCTGTGCGCTGGCCAGACAGTTATGCACAGGCCAGGCGGGTTTTAAGAGTTTTAATAAGTTTTAAAGAGTTTTAGGC
GGAAAAATCGCCTTTTTTCTCTTTTATATCAGTCACTTACATGTGTGACCGGTTCCCAATGTACGGCTTTGGGTTCCCAATGTACGGGTTCCGGTTC
CCAATGTACGGCTTTGGGTTCCCAATGTACGTGCTATCCACAGGAAAGAGACCTTTTCGACCTTTTTCCCCTGCTAGGGCAATTTGCCCTAGCATCT
GCTCCGTACATTAGGAACCGGCGGATGCTTCGCCCTCGATCAGGTTGCGGTAGCGCATGACTAGGATCGGGCCAGCCTGCCCCGCCTCCTCCTTCAA
ATCGTACTCCGGCAGGTCATTTGACCCGATCAGCTTGCGCACGGTGAAACAGAACTTCTTGAACTCTCCGGCGCTGCCACTGCGTTCGTAGATCGTC
TTGAACAACCATCTGGCTTCTGCCTTGCCTGCGGCGCGGCGTGCCAGGCGGTAGAGAAAACGGCCGATGCCGGGATCGATCAAAAAGTAATCGGGGT
GAACCGTCAGCACGTCCGGGTTCTTGCCTTCTGTGATCTCGCGGTACATCCAATCAGCTAGCTCGATCTCGATGTACTCCGGCCGCCCGGTTTCGCT
CTTTACGATCTTGTAGCGGCTAATCAAGGCTTCACCCTCGGATACCGTCACCAGGCGGCCGTTCTTGGCCTTCTTCGTACGCTGCATGGCAACGTGC
GTGGTGTTTAACCGAATGCAGGTTTCTACCAGGTCGTCTTTCTGCTTTCCGCCATCGGCTCGCCGGCAGAACTTGAGTACGTCCGCAACGTGTGGAC
GGAACACGCGGCCGGGCTTGTCTCCCTTCCCTTCCCGGTATCGGTTCATGGATTCGGTTAGATGGGAAACCGCCATCAGTACCAGGTCGTAATCCCA
CACACTGGCCATGCCGGCCGGCCCTGCGGAAACCTCTACGTGCCCGTCTGGAAGCTCGTAGCGGATCACCTCGCCAGCTCGTCGGTCACGCTTCGAC
AGACGGAAAACGGCCACGTCCATGATGCTGCGACTATCGCGGGTGCCCACGTCATAGAGCATCGGAACGAAAAAATCTGGTTGCTCGTCGCCCTTGG
GCGGCTTCCTAATCGACGGCGCACCGGCTGCCGGCGGTTGCCGGGATTCTTTGCGGATTCGATCAGCGGCCGCTTGCCACGATTCACCGGGGCGTGC
TTCTGCCTCGATGCGTTGCCGCTGGGCGGCCTGCGCGGCCTTCAACTTCTCCACCAGGTCATCACCCAGCGCCGCGCCGATTTGTACCGGGCCGGAT
GGTTTGCGACCGTCACGCCGATTCCTCGGGCTTGGGGGTTCCAGTGCCATTGCAGGGCCGGCAGACAACCCAGCCGCTTACGCCTGGCCAACCGCCC
GTTCCTCCACACATGGGGCATTCCACGGCGTCGGTGCCTGGTTGTTCTTGATTTTCCATGCCGCCTCCTTTAGCCGCTAAAATTCATCTACTCATTT
ATTCATTTGCTCATTTACTCTGGTAGCTGCGCGATGTATTCAGATAGCAGCTCGGTAATGGTCTTGCCTTGGCGTACCGCGTACATCTTCAGCTTGG
TGTGATCCTCCGCCGGCAACTGAAAGTTGACCCGCTTCATGGCTGGCGTGTCTGCCAGGCTGGCCAACGTTGCAGCCTTGCTGCTGCGTGCGCTCGG
ACGGCCGGCACTTAGCGTGTTTGTGCTTTTGCTCATTTTCTCTTTACCTCATTAACTCAAATGAGTTTTGATTTAATTTCAGCGGCCAGCGCCTGGA
CCTCGCGGGCAGCGTCGCCCTCGGGTTCTGATTCAAGAACGGTTGTGCCGGCGGCGGCAGTGCCTGGGTAGCTCACGCGCTGCGTGATACGGGACTC
AAGAATGGGCAGCTCGTACCCGGCCAGCGCCTCGGCAACCTCACCGCCGATGCGCGTGCCTTTGATCGCCCGCGACACGACAAAGGCCGCTTGTAGC
CTTCCATCCGTGACCTCAATGCGCTGCTTAACCAGCTCCACCAGGTCGGCGGTGGCCCATATGTCGTAAGGGCTTGGCTGCACCGGAATCAGCACGA
AGTCGGCTGCCTTGATCGCGGACACAGCCAAGTCCGCCGCCTGGGGCGCTCCGTCGATCACTACGAAGTCGCGCCGGCCGATGGCCTTCACGTCGCG
GTCAATCGTCGGGCGGTCGATGCCGACAACGGTTAGCGGTTGATCTTCCCGCACGGCCGCCCAATCGCGGGCACTGCCCTGGGGATCGGAATCGACT
AACAGAACATCGGCCCCGGCGAGTTGCAGGGCGCGGGCTAGATGGGTTGCGATGGTCGTCTTGCCTGACCCGCCTTTCTGGTTAAGTACAGCGATAA
CCTTCATGCGTTCCCCTTGCGTATTTGTTTATTTACTCATCGCATCATATACGCAGCGACCGCATGACGCAAGCTGTTTTACTCAAATACACATCAC
CTTTTTAGACGGCGGCGCTCGGTTTCTTCAGCGGCCAAGCTGGCCGGCCAGGCCGCCAGCTTGGCATCAGACAAACCGGCCAGGATTTCATGCAGCC
GCACGGTTGAGACGTGCGCGGGCGGCTCGAACACGTACCCGGCCGCGATCATCTCCGCCTCGATCTCTTCGGTAATGAAAAACGGTTCGTCCTGGCC
GTCCTGGTGCGGTTTCATGCTTGTTCCTCTTGGCGTTCATTCTCGGCGGCCGCCAGGGCGTCGGCCTCGGTCAATGCGTCCTCACGGAAGGCACCGC
GCCGCCTGGCCTCGGTGGGCGTCACTTCCTCGCTGCGCTCAAGTGCGCGGTACAGGGTCGAGCGATGCACGCCAAGCAGTGCAGCCGCCTCTTTCAC
GGTGCGGCCTTCCTGGTCGATCAGCTCGCGGGCGTGCGCGATCTGTGCCGGGGTGAGGGTAGGGCGGGGGCCAAACTTCACGCCTCGGGCCTTGGCG
GCCTCGCGCCCGCTCCGGGTGCGGTCGATGATTAGGGAACGCTCGAACTCGGCAATGCCGGCGAACACGGTCAACACCATGCGGCCGGCCGGCGTGG
TGGTGTCGGCCCACGGCTCTGCCAGGCTACGCAGGCCCGCGCCGGCCTCCTGGATGCGCTCGGCAATGTCCAGTAGGTCGCGGGTGCTGCGGGCCAG
GCGGTCTAGCCTGGTCACTGTCACAACGTCGCCAGGGCGTAGGTGGTCAAGCATCCTGGCCAGCTCCGGGCGGTCGCGCCTGGTGCCGGTGATCTTC
TCGGAAAACAGCTTGGTGCAGCCGGCCGCGTGCAGTTCGGCCCGTTGGTTGGTCAAGTCCTGGTCGTCGGTGCTGACGCGGGCATAGCCCAGCAGGC
CAGCGGCGGCGCTCTTGTTCATGGCGTAATGTCTCCGGTTCTAGTCGCAAGTATTCTACTTTATGCGACTAAAACACGCGACAAGAAAACGCCAGGA

Supplementary Sequence S2. The DNA sequence of the binary vector pCGS710 provided by Drs. Daniel Voytas and Colby Starker (continued).

AAAGGGCAGGGCGGCAGCCTGTCGCGTAACTTAGGACTTGTGCGACATGTCGTTTTCAGAAGACGGCTGCACTGAACGTCAGAAGCCGACTGCACTA
TAGCAGCGGAGGGGTTGGATCAAAGTACTTTGATCCCGAGGGGAACCCTGTGGTTGGCATGCACATACAAATGGACGAACGGATAAACCTTTTCACG
CCCTTTTAAATATCCGTTATTCTAATAAACGCTCTTTTCTCTTAGGTTTACCCGCCAATATATCCTGTCAAACACTGATAGTTTAAACTGAAGGCGG
GAAACGACAATCTGATCCAAGCTCAAGCTGCTCTAGCATTCGCCATTCAGGCTGCGCAACTGTTGGGAAGGGCGATCGGTGCGGGCCTCTTCGCTAT
TACGCCAGCTGGCGAAAGGGGGATGTGCTGCAAGGCGATTAAGTTGGGTAACGCCAGGGTTTTCCCAGTCACGACGTTGTAAAACGACGGCCAGTGC
CAAGCTTGGCGTGCCTGCAGGTCAACATGGTGGAGCACGACACACTTGTCTACTCCAAAAATATCAAAGATACAGTCTCAGAAGACCAAAGGGCAAT
TGAGACTTTTCAACAAAGGGTAATATCCGGAAACCTCCTCGGATTCCATTGCCCAGCTATCTGTCACTTTATTGTGAAGATAGTGGAAAAGGAAGGT
GGCTCCTACAAATGCCATCATTGCGATAAAGGAAAGGCCATCGTTGAAGATGCCTCTGCCGACAGTGGTCCCAAAGATGGACCCCCACCCACGAGGA
GCATCGTGGAAAAAGAAGACGTTCCAACCACGTCTTCAAAGCAAGTGGATTGATGTGATAACATGGTGGAGCACGACACACTTGTCTACTCCAAAAA
TATCAAAGATACAGTCTCAGAAGACCAAAGGGCAATTGAGACTTTTCAACAAAGGGTAATATCCGGAAACCTCCTCGGATTCCATTGCCCAGCTATC
TGTCACTTTATTGTGAAGATAGTGGAAAAGGAAGGTGGCTCCTACAAATGCCATCATTGCGATAAAGGAAAGGCCATCGTTGAAGATGCCTCTGCCG
ACAGTGGTCCCAAAGATGGACCCCCACCCACGAGGAGCATCGTGGAAAAAGAAGACGTTCCAACCACGTCTTCAAAGCAAGTGGATTGATGTGATAT
CTCCACTGACGTAAGGGATGACGCACAATCCCACTATCCTTCGCAAGACCCTTCCTCTATATAAGGAAGTTCATTTCATTTGGAGAGGACCTCGACT
CTAGAGGATCCCCGGGTACCGGGCCCCCCCTCGAGGCGCGCCAAGCTATCAAACAAGTTTGTACAAAAAAGCTGAACGAGAAACGTAAAATGATATA
AATATCAATATATTAAATTAGATTTTGCATAAAAAACAGACTACATAATACTGTAAAACACAACATATCCAGTCACTATGGCGGCCGCATTAGGCAC
CCCAGGCTTTACACTTTATGCTTCCGGCTCGTATAATGTGTGGATTTTGAGTTAGGATCCGGCGAGATTTTCAGGAGCTAAGGAAGCTAAAATGGAG
AAAAAAATCACTGGATATACCACCGTTGATATATCCCAATGGCATCGTAAAGAACATTTTGAGGCATTTCAGTCAGTTGCTCAATGTACCTATAACC
AGACCGTTCAGCTGGATATTACGGCCTTTTTAAAGACCGTAAAGAAAAATAAGCACAAGTTTTATCCGGCCTTTATTCACATTCTTGCCCGCCTGAT
GAATGCTCATCCGGAATTCCGTATGGCAATGAAAGACGGTGAGCTGGTGATATGGGATAGTGTTCACCCTTGTTACACCGTTTTCCATGAGCAAACT
GAAACGTTTTCATCGCTCTGGAGTGAATACCACGACGATTTCCGGCAGTTTCTACACATATATTCGCAAGATGTGGCGTGTTACGGTGAAAACCTGG
CCTATTTCCCTAAAGGGTTTATTGAGAATATGTTTTTCGTCTCAGCCAATCCCTGGGTGAGTTTCACCAGTTTTGATTTAAACGTGGCCAATATGGA
CAACTTCTTCGCCCCCGTTTTCACCATGGGCAAATATTATACGCAAGGCGACAAGGTGCTGATGCCGCTGGCGATTCAGGTTCATCATGCCGTCTGT
GATGGCTTCCATGTCGGCAGAATGCTTAATGAATTACAACAGTACTGCGATGAGTGGCAGGGCGGGGCGTAATCTAGAGGATCCGGCTTACTAAAAG
CCAGATAACAGTATGCGTATTTGCGCGCTGATTTTTGCGGTATAAGAATATATACTGATATGTATACCCGAAGTATGTCAAAAAGAGGTGTGCTATG
AAGCAGCGTATTACAGTGACAGTTGACAGCGACAGCTATCAGTTGCTCAAGGCATATATGATGTCAATATCTCCGGTCTGGTAAGCACAACCATGCA
GAATGAAGCCCGTCGTCTGCGTGCCGAACGCTGGAAAGCGGAAAATCAGGAAGGGATGGCTGAGGTCGCCCGGTTTATTGAAATGAACGGCTCTTTT
GCTGACGAGAACAGGGACTGGTGAAATGCAGTTTAAGGTTTACACCTATAAAAGAGAGAGCCGTTATCGTCTGTTTGTGGATGTACAGAGTGATATT
ATTGACACGCCCGGGCGACGGATGGTGATCCCCCTGGCCAGTGCACGTCTGCTGTCAGATAAAGTCTCCCGTGAACTTTACCCGGTGGTGCATATCG
GGGATGAAAGCTGGCGCATGATGACCACCGATATGGCCAGTGTGCCGGTCTCCGTTATCGGGGAAGAAGTGGCTGATCTCAGCCACCGCGAAAATGA
CATCAAAAACGCCATTAACCTGATGTTCTGGGGAATATAAATGTCAGGCTCCCTTATACACAGCCAGTCTGCAGGTCGACCATAGTGACTGGATATG
TTGTGTTTTACAGTATTATGTAGTCTGTTTTTTATGCAAAATCTAATTTAATATATTGATATTTATATCATTTTACGTTTCTCGTTCAGCTTTCTTG
TACAAAGTGGTTCGATAATTCTTAATTAACTAGTTCTAGAGCGGCCGCCACCGCGGTGGAGCTCGAATTTCCCCGATCGTTCAAACATTTGGCAATA
AAGTTTCTTAAGATTGAATCCTGTTGCCGGTCTTGCGATGATTATCATATAATTTCTGTTGAATTACGTTAAGCATGTAATAATTAACATGTAATGC
ATGACGTTATTTATGAGATGGGTTTTTATGATTAGAGTCCCGCAATTATACATTTAATACGCGATAGAAAACAAAATATAGCGCGCAAACTAGGATA
AATTATCGCGCGCGGTGTCATCTATGTTACTGAATTCGTAATCATGGTCATAGAAGCTTGCATGCCTGCAGGTCGACTCTAGAGGATCCCCGGGTAC
CGAGCTCGAATTCGTAATCATGTCATAGCTGTTTCCTGTGTGAAATTGTTATCCGCTCACAATTCCACACAACATACGAGCCGGAAGCATAAAGTGT
AAAGCCTGGGGTGCCTAATGAGTGAGCTAACTCACATTAATTGCGTTGCGCTCACTGCCCGCTTTCCAGTCGGGAAACCTGTCGTGCCAGCTGCATT
AATGAATCGGCCAACGCGCGGGGAGAGGCGGTTTGCGTATTGGCTAGAGCAGCTTGCCAACATGGTGGAGCACGACACTCTCGTCTACTCCAAGAAT
ATCAAAGATACAGTCTCAGAAGACCAAAGGGCTATTGAGACTTTTCAACAAAGGGTAATATCGGGAAACCTCCTCGGATTCCATTGCCCAGCTATCT
GTCACTTCATCAAAAGGACAGTAGAAAAGGAAGGTGGCACCTACAAATGCCATCATTGCGATAAAGGAAAGGCTATCGTTCAAGATGCCTCTGCCGA
CAGTGGTCCCAAAGATGGACCCCCACCCACGAGGAGCATCGTGGAAAAAGAAGACGTTCCAACCACGTCTTCAAAGCAAGTGGATTGATGTGATAAC
ATGGTGGAGCACGACACTCTCGTCTACTCCAAGAATATCAAAGATACAGTCTCAGAAGACCAAAGGGCTATTGAGACTTTTCAACAAAGGGTAATAT
CGGGAAACCTCCTCGGATTCCATTGCCCAGCTATCTGTCACTTCATCAAAAGGACAGTAGAAAAGGAAGGTGGCACCTACAAATGCCATCATTGCGA
TAAAGGAAAGGCTATCGTTCAAGATGCCTCTGCCGACAGTGGTCCCAAAGATGGACCCCCACCCACGAGGAGCATCGTGGAAAAAGAAGACGTTCCA
ACCACGTCTTCAAAGCAAGTGGATTGATGTGATATCTCCACTGACGTAAGGGATGACGCACAATCCCACTATCCTTCGCAAGACCTTCCTCTATATA
AGGAAGTTCATTTCATTTGGAGAGGACACGCTGAAATCACCAGTCTCTCTCTACAAATCTATCTCTCTCGAGCTTTCGCAGATCTGTCGATCGACCA
TGGGGATTGAACAAGATGGATTGCACGCAGGTTCTCCGGCCGCTTGGGTGGAGAGGCTATTCGGCTATGACTGGGCACAACAGACAATCGGCTGCTC
TGATGCCGCCGTGTTCCGGCTGTCAGCGCAGGGGCGCCCGGTTCTTTTTGTCAAGACCGACCTGTCCGGTGCCCTGAATGAACTCCAGGACGAGGCA
GCGCGGCTATCGTGGCTGGCCACGACGGGCGTTCCTTGCGCAGCTGTGCTCGACGTTGTCACTGAAGCGGGAAGGGACTGGCTGCTATTGGGCGAAG
TGCCGGGGCAGGATCTCCTGTCATCTCACCTTGCTCCTGCCGAGAAAGTATCCATCATGGCTGATGCAATGCGGCGGCTGCATACGCTTGATCCGGC
TACCTGCCCATTCGACCACCAAGCGAAACATCGCATCGAGCGAGCACGTACTCGGATGGAAGCCGGTCTTGTCGATCAGGATGATCTGGACGAAGAG
CATCAGGGGCTCGCGCCAGCCGAACTGTTCGCCAGGCTCAAGGCGCGCATGCCCGACGGCGAGGATCTCGTCGTGACACATGGCGATGCCTGCTTGC
CGAATATCATGGTGGAAAATGGCCGCTTTTCTGGATTCATCGACTGTGGCCGGCTGGGTGTGGCGGACCGCTATCAGGACATAGCGTTGGCTACCCG
TGATATTGCTGAAGAGCTTGGCGGCGAATGGGCTGACCGCTTCCTCGTGCTTTACGGTATCGCCGCTCCCGATTCGCAGCGCATCGCCTTCTATCGC
CTTCTTGACGAGTTCTTCTGAGCGGGACTCTGGGGTTCGGATCGATCCTCTAGCTAGAGTCGATCGACAAGCTCGAGTTTCTCCATAATAATGTGTG
AGTAGTTCCCAGATAAGGGAATTAGGGTTCCTATAGGGTTTCGCTCATGTGTTGAGCATATAAGAAACCCTTAGTATGTATTTGTATTTGTAAAATA
CTTCTATCAATAAAATTTCTAATTCCTAAAACCAAAATCCAGTACTAAAATCCAGATCCCCCGAATTAATTCGGCGTTAATTCAGTACATTAAAAAC
GTCCGCAATGTGTTATTAAGTTGTCTAAGCGTCAATTTGTTTACACCACAATATATCCTGCCA

July 2023

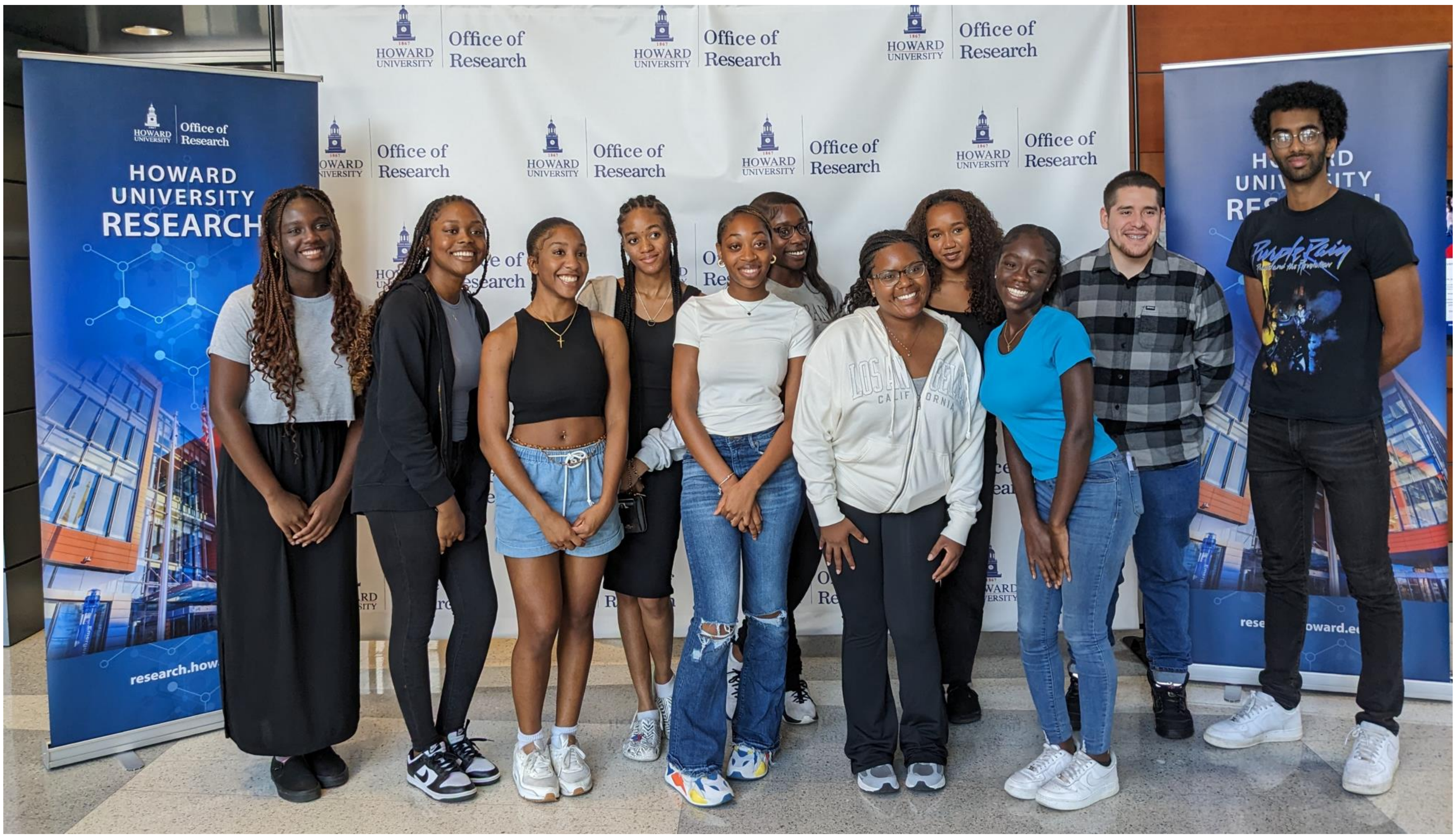

July 2024

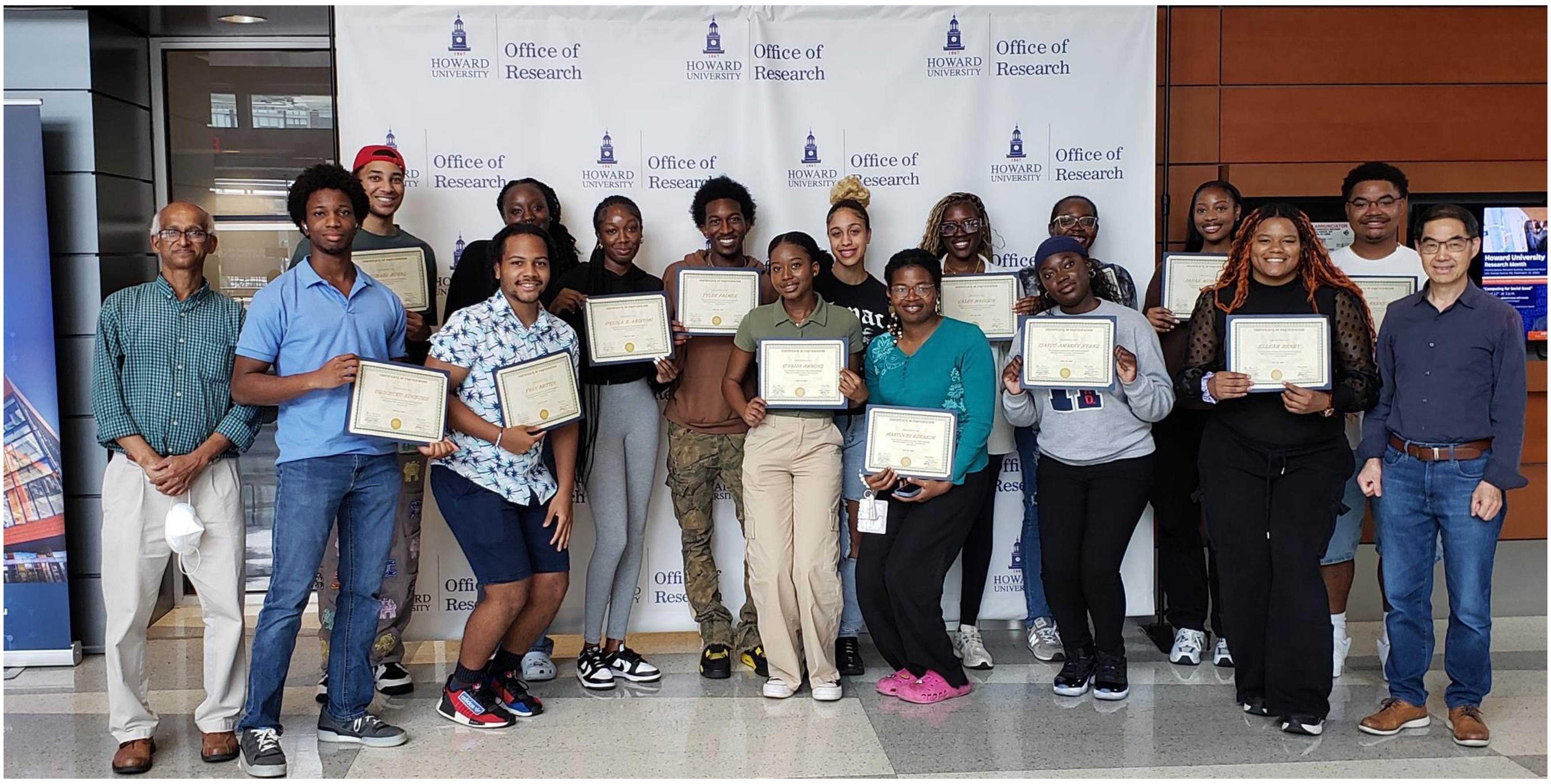

Supplementary Figure S1. Group photos of undergraduate students participated in the two summer CRISPR workshops.

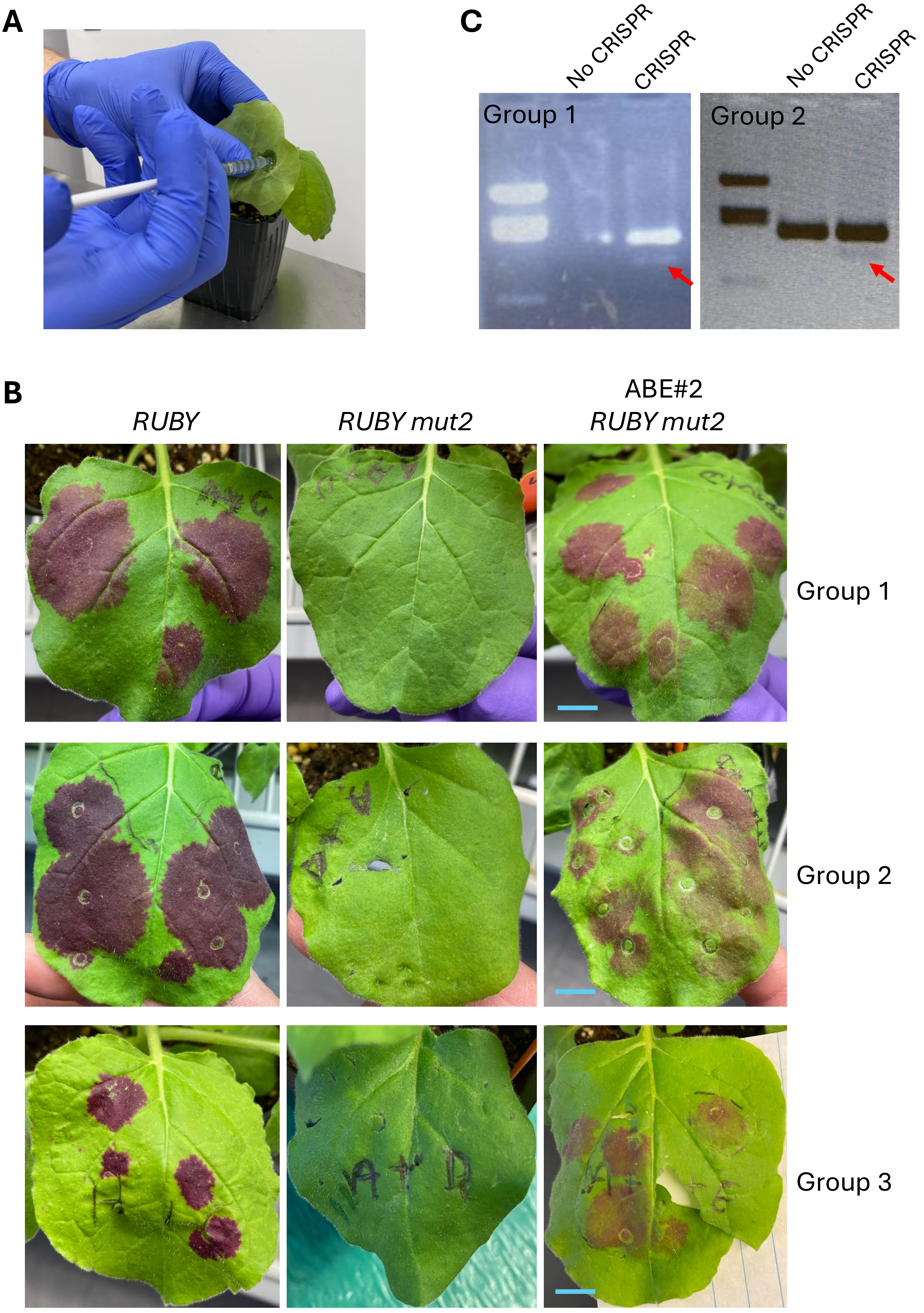

Supplementary Figure S2. Students' success with CRISPR/Cas9-enabled gene editing. A, Demonstration of agroinfiltration of leaves of *Nicotiana benthamiana*. B, Selected photos taken at 4.5 days post-agroinfiltration, demonstrating successful correction of a nonsense mutation (415**T**AG → 415**C**AG) in *RUBYmut2* by ABE#2. C, Gel photos showing PCR amplification of wild-type *GFP* and mutated *GFP* with large deletions (red arrows). Bar = 2 cm, which is applicable to all photos.

Supplementary Table S1. Plant materials, agrobacterial strains, primers and other key reagents

Name of materials	Purpose	Quantity
Nicotiana benthamiana wild-type	For agroinfiltration	6-12 plants
N. benthamiana 16c (GFP)	For agroinfiltration	6-12 plants
Agrobacterium GV3101_TCV-CP	For suppessing transgene silencing	50 ml
Agrobacterium GV3101_RUBY	Reporting success of transient expression	15 ml
Agrobacterium GV3101_RUBY mutant1	Target for base-editing	15 ml
Agrobacterium GV3101_RUBY mutant2	Target for base-editing	15 ml
Agrobacterium GV3101_RUBY mutant3	Target for base-editing	15 ml
Agrobacterium GV3101_RUBY mutant4	Target for base-editing	15 ml
Agrobacterium GV3101_ABE#1 (pYPQ5236)	Base-editing	15 ml
Agrobacterium GV3101_ABE#2 (pYPQ5237)	Base-editing	15 ml
Agrobacterium GV3101_ABE#3 (pYPQ5238)	Base-editing	15 ml
Agrobacterium GV3101_ABE#4 (pYPQ5239)	Base-editing	15 ml
Agrobacterium GV3101_Cas9+4sgRNA /pDGE643	Targeted mutagenesis of GFP	15 ml
I ml syringes	For agroinfiltration	20
Face Shields	For face protection while injcting agro cells	6
pLR3850 plasmid	For cloning sgRNA	1000 ng
Bsa I-HF restrictive enzyme (NEB)	Golden Gate cloning	20 µl
T4 DNA ligase (NEB)	DNA ligation	20 µl
Nhe I restrictive enzyme (NEB)	Cuting GFP	20 µl
Dde I restrictive enzyme (NEB)	Cutting GFP	20 µl
Taq polymerase	PCR	20 µl
dNTPs (2mM)	PCR	200 µl
GelRed (Biotium, #41003T)	Staining DNA in gel	0.1 ml
E coli competent cell (DH5α)	Cloning	500 µl
GFP-gRNA: attGAGAAAGTAGTGACAAGTGT	Cloning sgRNA	20 µl (100 µM)
GFP-gRNA2: attGATACCCAGATCATATGAAG	For cloning sgRNA	20 µl (100 µM)
GFP-gRNA3: attgAAGCTCGATCCTGTTGACGA	For cloning sgRNA	20 µl (100 µM)
GFP-gRNA4: attgTAAGGGAATCGATTTCAAGG	For cloning sgRNA	20 µl (100 µM)
GFP-F: caccATGAAGACTAATCTTTTTCTCTTTCT	For PCR	20 µl (100 µM)
GFP-R: ctcTTAAAGCTCATCATGTTTGTA	For PCR	20 µl (100 µM)
NucleoSpin Plant II kit (Takara Bio)	For genomic DNA extraction	1 kit

Supplementary Information S1

Protocol #1: Transient gene expression in *Nicotiana benthamiana* via agroinfiltration

Materials

- Yeast extract beef (YEB) medium (solid and liquid): 5 g/L beef extract, 1 g/L yeast extract, 5 g/L peptone, 5 g/L sucrose, 0.5 g/L $MgCl_2$, 20 g/L agar (solid media only), pH 7.0
- Antibiotics (final concentration): rifampicin (R) (25 µg/mL), gentamycin (G) (25 µg/mL), kanamycin (K) (50 µg/mL), spectinomycin (S) (100 µg/mL)
- *Agrobacterium* strains (antibiotic selection):
    A. *TCV-CP* (R/G/S): for suppressing transgene silencing
    B. *GFP-gRNA* (R/G/S): CRISPR construct for GFP mutagenesis
    C. *WT RUBY* (R/G/S): functional *RUBY*
    D. *RUBY mutant #2* (R/G/S): non-functional *RUBY* containing a CAG to TAG mutation in *CYP76AD1*
    E. *ABE # 2* (*pYPQ5237*) (R/G/K): CRISPR construct containing an adenine base editor (ABE) and a sgRNA targeting the mutation in *RUBY mutant #2*
- Infiltration buffer: 10 mM $MgCl_2$, 10 mM MES, 200 µM acetosyringone, pH 5.6
    - Add acetosyringone just before the buffer is used. It can be prepared as a 100 mM stock.
- *Nicotiana benthamiana* WT and 16c plants (4-6 weeks old)
- 1 mL needleless syringes
- Petri dishes
- Test tubes with caps
- Inoculation loops
- 15 mL centrifuge tubes
- 28 °C incubator
- 28 °C shaking incubator
- Centrifuge
- Vortexer
- Spectrophotometer and cuvettes
- Face shields or safety glasses
- Marker pens and labels

Method

Day -3 (3 days before the workshop)

1. To obtain fresh colony matter, streak out the *Agrobacterium* strains (colony matter or glycerol stocks) on YEB plates containing the appropriate antibiotics and incubate them at 28 °C for two days.
    - This step may be done beforehand by a TA, technician, etc.

Day -1

2. Scrape up a fresh colony and inoculate 5-6 mL YEB with the appropriate antibiotics. Incubate overnight at 28 °C and 200 rpm.
    - More *TCV-CP* will be used than the other *Agrobacterium* strains, so we suggest preparing 2-3 times more of this strain.
    - This step may be done beforehand by a TA, technician, etc.

Day 1

3. Transfer the overnight cultures to 15 mL centrifuge tubes and centrifuge at 4000 g for 10 min.
4. Discard the supernatants and resuspend the pellets in 5-6 mL infiltration buffer by vortexing.
5. Measure the OD_{600} of each cell suspension and dilute them to OD_{600} = 0.5 with infiltration buffer.
6. Prepare the cell suspension mixtures for infiltration using equal volumes of each *Agrobacterium* strain listed.
    - A + B, for infiltration into *N. benthamiana* 16c
    - A alone, for infiltration into *N. benthamiana* 16c
    - A + C, for infiltration into *N. benthamiana* WT
    - A+ D, for infiltration into *N. benthamiana* WT
    - A + D + E, for infiltration into *N. benthamiana* WT
7. Using a needless 1 mL syringe, suck up some of a mixture and infiltrate it into 2-3 differently aged leaves of the indicated *N. benthamiana* line.
    - To infiltrate the leaves, gently place the opening of the syringe against the underside of the leaf away from major veins and slowly press down on the plunger while applying gently pressure on the other side of the leaf with your finger.
    - Use a separate syringe for each mixture.
    - Wear a face shield or safety glasses while infiltrating the leaves.
8. Use a marker pen to label and mark the infiltrated area of the leaf while being careful not to damage the plant tissue.
9. Place the plants in the dark and maintain high relative humidity overnight.
10. Return the plants to normal growth conditions for 3-4 days.
11. If an epifluorescent microscope or confocal microscope is available, it can be used to visualize a likely reduction of the GFP fluorescence in the leaf tissues agroinfiltrated with the CRISPR construct targeting GFP (i.e., A+B) relative to those with the negative control (i.e., A alone) at 3-4 days after agroinfiltration. Regardless of the outcome, this step offers students an opportunity to “see” GFP fluorescence with the aid of an appropriate microscope.

Supplementary Information S2

Protocol #2: CRISPR-enabled base-editing of *RUBY* in *Nicotiana benthamiana*

Materials

- All-in-one vector pLR3850
- Oligonucleotides:
  - RUBY-sgRNA-F: ggtcaCTCTATGGGGAGAGCAGATGCA
  - RUBY-sgRNA-R: aaacTGCATCTGCTCTCCCCATAGAGt
  - 165-F8: ctggaggcgaagggctacaa?
- *Escherichia coli* DH5α competent cells
- LB broth medium (Thermo Fisher Scientific)
- LB solid plates with Kanamycin (50 µg/mL)
- 0.1 M isopropyl β-D-1-thiogalactopyranoside (IPTG; Thermo Fisher)
- 20 mg/ml 5-bromo-4-chloro-3-indolyl β-D-galactopyranoside (X-gal; Thermo Fisher)
- *Bsa*I-HF®v2 (New England Biolabs)
- T4 DNA ligase and 10x T4 ligase buffer (New England Biolabs)
- *Taq* polymerase and corresponding buffer
- 10 mM deoxynucleotides (dNTP, New England Biolabs)
- PCR grade water
- 0.2 mL PCR tubes
- Agarose
- 1x TAE buffer
- 1 kb DNA ladder
- Biotium GelRed (for staining DNA in gel)
- 42 °C heating block or water bath
- 37 °C stationary and shaking incubators
- Thermocycler
- Plating beads (ColiRollersTM Plating Beads, Millipore Sigma) or spreaders
- Electrophoresis apparatus

Method

Day 1. Phosphorylation and annealing of sgRNAs

1. Thaw the oligos and other reagents on ice.
2. Prepare the reaction mixture in a PCR tube using the recipe below.

Component	Volume
RUBY-sgRNA-F (100 µM)	1.0 µL
RUBY-sgRNA-R (100 µM)	1.0 µL
T4 Polynucleotide Kinase (10 U/µL)	0.2 µL
T4 Polynucleotide Kinase buffer (10x)	1.0 µL
PCR Grade Water	6.8 µL
Total	10.0 µL

3. Phosphorylate and anneal the oligos using the following thermocycler program: 37 °C for 30 min; 95 °C for 5 min; ramp down to 25 °C at 5 °C min^{-1}.
   - Alternatively, cool down in boiled water instead of ramping down.
4. Store the phosphorylated and annealed oligos at -20 °C.

Day 2. Golden Gate Cloning of sgRNA into the all-one-one ABE vector

5. Assemble the sgRNA with the pLR3850 using the Golden Gate recipe below.

Component	Volume
T4 ligase buffer (10x)	2.0 µL
T4 DNA ligase	1.0 µL
*Bsa*I-HF®v2	1.0 µL
Annealed sgRNA oligos	2.0 uL
pLR3850	100 ng
PCR Grade Water	up to 20 µL
Total	20.0 µL

6. Incubate the reactions in a thermocycler with the following program.

Temperature (℃)	Time (min)	Cycles
37	5	10
16	10	
50	5	1
80	5	1
10	∞	n/a

7. *E. coli* transformation
   - Thaw DH5α competent cells on ice.
   - Add all mixture of the ligation reaction to the cells and mix by finger-flicking. Do not vortex the tube.
   - Incubate the tube on ice for 10 min. Do not mix.
   - Heat shock at 42 °C for 50 seconds, return tube to ice for 2 min.
   - Add 800 µL plain LB media to the tube and incubate for one hour at 37 °C with rotation or shaking (200–250 rpm).

- Add 70 µL 0.1 M IPTG and 70 µL 20 mg/mL X-gal to the surface of an LB plate supplemented with 50 µg/mL Kanamycin.
- Spread 100 µL of the *E.coli* on the selection plate and incubate overnight at 37 °C.

Day 3. Observation of blue-white selection and colony PCR

8. Pick a single colony with a sterile pipette tip and swirl in 10 µL of sterile water. Pick 3 white colonies and 2 blue colonies (as negative control) in total to test. Additionally, the positive plasmid is used as a positive control and water as another negative control.
9. Prepare the PCR reaction mixture as follows.

Component	Volume (1 reaction)	Volume (8x mix)
PCR Grade Water	17.8 µL	142.8 µL
Standard *Taq* Reaction Buffer (10x)	2.5 µL	20.0 µL
dNTPs (10 mM)	0.5 µL	4.0 µL
165-F8 (5 µM) (Forward Primer)	1.0 µL	8.0 µL
sgRNA-R (5 µM) (Reverse Primer)	1.0 µL	8.0 µL
Taq DNA Polymerase (5 U/µL)	0.2 µL	1.6 µL
Template DNA (bacteria-water suspension)	2.0 µL	n/a
Total	25.0 µL	

- Length of PCR product should be roughly 1700 bp

10. Incubate the reactions in a thermocycler with the following program.

Temperature	Time	Cycles
95 °C	30 sec	1
95 °C	30 sec	
57 °C	30 sec	34
68 °C	1 min and 40 sec	
68 °C	5 min	1
10 °C	∞	n/a

11. Store the PCR reactions at -20 °C overnight.

Day 4. Electrophoresis of colony PCR products

12. Add 15 µL (or more) of each sample to separate wells in a 1% agarose gel (in 1xTAE buffer with 1x GelRed (Biotium, 10000x; cat#: 41003).
13. Add 5 µL of 1kb DNA ladder to at least one well per row of wells.
14. Perform gel electrophoresis at 100-120V for ~40 min.
15. Visualize DNA bands using a UV transilluminator.

Supplementary Information S3

Protocol #3: Detection of CRISPR-induced deletions via PCR

Materials

- Agroinfiltrated *Nicotiana benthamiana* 16c leaves from Protocol #1
- NucleoSpin Plant II kit (Takara Bio)
- Bamboo toothpicks (or 10 µL tips)
- Dry ice
- PCR grade water
- CutSmart buffer (10x)
- *Nde*I restriction enzyme (20 U/µL)
- *Dde*I restriction enzyme (10 U/µL)
- *Taq* polymerase and corresponding buffer
- 10 mM deoxynucleotides (dNTP, New England Biolabs)
- Oligonucleotides:
    - GFP-F: ATGAAGACTAATCTTTTTCTCTTTCT
    - GFP-R: TTAAAGCTCATCATGTTTGTA
- Uncut genomic DNA prepared from leaf tissues subjected to CRIPSR mutagenesis by trainers as **positive control-1** (optional)
- *Nde*I & *Dde*I digested genomic DNA from leaf tissues subjected to CRIPSR mutagenesis prepared by trainers as **positive control-2** (optional)
- Agarose
- 1x TAE buffer
- 1 kb DNA ladder
- Biotium GelRed (for staining DNA in gel)
- Electrophoresis apparatus
- Nanodrop
- Thermocycler
- UV transilluminator

Method

Genomic DNA (gDNA) Extraction

(Using a NucleoSpin Plant II kit)

1. Harvest up to 100 mg leaf tissue and place in a 1.5 mL microtube.
    - ~25 mg should be sufficient
2. Incubate the tube on dry ice for 5-10 min to freeze the leaf tissue.
3. Use a bamboo toothpick with a flat end to grind the frozen leaf tissue into a powder inside of the microtube.

4. Add 400 µL PL1 and 5 µL RNase (20 mg/mL) to the microtube.
5. Vortex the tube and incubate at 60-65 °C for 10 min.
    - The elution buffer (PE) can also be incubated at 60-65 °C until the elution step.
6. Load the lysate onto a violet ring filter inside of a collection tube.
7. Centrifuge at 11,000 g for 2 min. Discard filter.
8. Add 450 µL PC to the flow through and pipet up and down to mix.
    - Alternatively, the mixture can be mixed by transferring it to a clean microtube and gently tapping it.
9. Load ≤ 700 µL of the sample onto a green ring column inside of a collection tube.
10. Centrifuge at 11,000 g for 1 min. Discard flow through.
11. Add 400 µL PW1 to the column.
12. Centrifuge at 11,000 g for 1 min. Discard flow through.
13. Add 700 µL PW2 to the column.
14. Centrifuge at 11,000 g for 1 min. Discard flow through.
15. Add 200 µL PW2 to the column.
16. Centrifuge at 11,000 g for 2 min. Discard flow through.
17. Transfer the column to a clean microtube.
18. Add 50 µL PE (60-65 °C) to the column and incubate at 60-65 °C for 5 min.
19. Centrifuge at 11,000 g for 2 min.
20. Measure the DNA concentration and quality using PE as a blank.

Restriction Digest of gDNA

21. Let reagents thaw. (Thaw heat sensitive reagents on ice.)
22. For the digestion treatment, prepare the master mix in a 1.5 mL tube.

**Component**	**Volume (1 reaction)**	**Volume (3x mix)**
PCR Grade Water	15.5 µL	46.5 µL
CutSmart Buffer (10x)	2.5 µL	7.5 µL
*Nde*I (20 U/µL)	1.0 µL	3.0 µL
*Dde*I (20 U/µL)	1.0 µL	3.0 µL
gDNA Extract	5.0 µL	n/a
Total	25.0 µL	

23. Gently tap the tube to mix, then dispense 20 µL of the master mix into two PCR tubes.
24. Add 5 µL gDNA from the CRISPR-treated tissue to one tube and 5 µL gDNA from the non-CRISPR-treated tissue to the other. The total amount of gDNA in each tube should be ~85 ng.
25. Gently tap the tubes to mix.
26. Incubate the digestion samples in a thermocycler at 37 °C for 1.5 hr and then at 65 °C for 20 min.

PCR for GFP

27. Let reagents thaw. (Thaw heat sensitive reagents on ice.)
28. Prepare PCR master mix for 8 by adding the following to a 1.5 mL tube.

Component	Volume (1 reaction)	Volume (8x mix)
PCR Grade Water	17.8 µL	142.4 µL
Standard *Taq* Reaction Buffer (10x)	2.5 µL	20.0 µL
dNTPs (10 mM)	0.5 µL	4.0 µL
GFP-F (5 µM) (Forward Primer)	1.0 µL	8.0 µL
GFP-R (5 µM) (Reverse Primer)	1.0 µL	8.0 µL
Taq DNA Polymerase (5 U/µL)	0.2 µL	1.6 µL
Template DNA	2.0 µL	n/a
Total	25.0 µL	

29. Gently tap the tube to mix, then dispense 23 µL into five PCR tubes.
30. Add the following to separate PCR tubes with 23 µL master mix.
    a. 2 µL original gDNA from non-CRISPR-treated tissue
    b. 2 µL original gDNA from CRISPR-treated tissue
    c. 2 µL digested gDNA from non-CRISPR-treated tissue
    d. 2 µL digested gDNA from CRISPR-treated tissue
    e. 2 µL Positive control-1 (optional)
    f. 2 µL Positive control-2 (optional)
    g. 2 µL PCR grade water (as a negative control)
31. Mix the samples by gently tapping the tubes.
32. Run the samples in the thermocycler using the following program.

Temperature	Time	Cycles
95 °C	30 sec	1
95 °C	30 sec	
50 °C	30 sec	34
68 °C	49 sec	
68 °C	5 min	1
10 °C	∞	n/a

    - The length of the PCR product should be roughly 800 bp.
33. Add 15 µL (or more) of each sample to separate wells in a 1% agarose gel (in TAE buffer with 1x GelRed (Biotium, 10000x; cat#: 41003).
34. Add 6 or 8 µL of 1 kb DNA ladder to at least one well per row of wells.
35. Perform gel electrophoresis at 100-120V for ~40 min.
36. Visualize DNA bands using a UV transilluminator.

Supplementary Information S4

A Daily Schedule for A Five-Day CRISPR Workshop

Day 1 / Theme 1: *Agrobacterium*-mediated transient expression

Day 1-Morning (9:30-12:00)

Lecture 1 (9:30-10:30): Plant biotechnology featuring *Agrobacterium*-mediated transformation

Lecture 2 (11:00-12:00): Plant genome editing by CRISPR-Cas systems

Lunch break

Day 1-Afternoon (2:00-5:00)

There are two experiments during this workshop. The order of experiments is adjusted to suit the week-long workshop schedule. Some steps are moved to the beginning of the workshop to ensure adequate time for transient expression of DNA constructs in plants. Students will be divided into 3 groups and each group will be supervised by a TA with help from co-PIs.

Brief Introduction of the the experiments (2:00-3:00):
CRISPR-targeted mutagenesis of the *GFP* transgene in *Nicotiana benthamiana* 16c
CRISPR-enabled base-editing of *RUBY* in *N. benthamiana*

Protocols used for the three experiments:
Protocol #1: *Agrobacterium*-mediated transient expression in *N. benthamiana*
Protocol #2: CRISPR-Cas9 ABE vector construction
Protocol #3: Detection of CRISPR-induced mutations via PCR

Experiment 1.1 (3:00-4:00): CRISPR/Cas9 mutagenesis of *GFP* - Agroinfiltration
Agrobacterial cells harboring a binary plasmid (pDGE463) that contains the expression cassettes for *Cas9* and four sgRNAs targeting *GFP* will be infiltrated into leaves of *N. benthamiana* stably expressing *GFP*. Five-week-old plants of *N. benthamiana* 16c, cell suspensions of agrobacterium strains containing the *Cas9-sgRNA/pDGE347* plasmid or the plasmid expressing *TCV-CP* (for suppressing transgene silencing) will be prepared just before the workshop. For experimental details, see Protocol #1.

Experiment 2.1 (4:00-5:00): CRISPR-enabled base-editing of *RUBY* - Agroinfiltration

Leave of wild-type *N. benthamiana* plants will be infiltrated with a mixture of agrobacterial cells from different strains: (1) those containing a plasmid for expressing the wild-type *RUBY* reporter as positive control, (2) those containing a plasmid for expressing a mutant *RUBY* (*RUBYm2*) reporter as negative control, (3) those containing a plasmid for expressing RUBYm2 + those containing a plasmid for expressing nCas9-deaminase fusion and a corresponding sgRNA (ABE#2). Five-week-old plants of *N. benthamiana*, cell suspensions of agrobacterium strains containing the plasmids for expressing *RUBY* or *RUBYm2*, or *ABE#2* or *TCV-CP* (for suppressing transgene silencing) will be prepared just before the workshop. For experimental details, see Protocol #1.

Day 2 / Theme 2: Molecular cloning

Day 2-Morning (9:30-12:00)

Lecture 3 (9:30-10:30): Molecular cloning made easy

Lecture 4 (11:00-12:00): How to design your CRISPR experiments

<u>Lunch break</u>

Day 2-Afternoon (2:00-5:00).

Experiment 2.2 (2:00-5:00): CRISPR-enabled base-editing of *RUBY* – Cloning of sgRNA
The sgRNA will be cloned into binary vector pLRQ3850 that contains both the *Cas9*-adenosine deaminase fusion gene and the *sgRNA* expression cassettes. For experimental details, see Protocol #2.

Day 3 / Theme 3: DNA Sequence Analysis

Day 3-Morning (9:30-12:00)

Lecture 5 (9:30-12:30): DNA sequence analysis using Benchling (https://www.benchling.com). This lecture includes Benchling registration, sequence downloading from NCBI, design of primers and sgRNAs, and sequence alignment and analysis.

<u>Lunch break</u>

Day 3-Afternoon (2:00-5:00)

Practice with Benchling (2:00-4:00): Every student will independently design a good sgRNA that specifically target the *FER* gene (AT3G51550) of Arabidopsis or any other genes of interest.

Experiment 2.3 (3:30-4:00): CRISPR-enabled base-editing of *RUBY* – Transfection of *E coli* cells.
Competent *E coli* cells will be transfected with the ligated products from the Golden Gate cloning reactions in Experiment 2.2 via heat shock according to Protocol #2.

Day 4 / Theme 4: DNA extraction, PCR and gel electrophoresis

Day 4-Morning (9:30-12:00)

Experiment 1.2 (9:30-11:00): CRISPR/Cas9 mutagenesis of *GFP* – DNA extraction
Genomic DNA will be extracted from leaf areas infiltrated with agrobacterial cells in Experiment 1.1. For experimental details, see Protocol #3.

Experiment 1.3 (11:00-12:00): CRISPR/Cas9 mutagenesis of *GFP* – Enzymatic digestion
Half of the genomic DNA from Experiment 1.2 will be subjected to digestion with restriction enzymes, *Nde*I and/or *Dde*I to eliminate the wild-type *GFP* gene by following Protocol #3.

Lunch break

Day 4-Afternoon (1:30-5:00)

Experiment 1.4 (1:30-3:30): CRISPR/Cas9 mutagenesis of *GFP* – PCR
The undigested and digested genomic DNA samples will be used for PCR amplification of *GFP* with gene-specific primers in a Thermocycler by following protocol #3. After PCR, save the PCR products at 4 °C for gel electrophoresis on Day 5.

Experiment 2.3 (2:00-5:00): CRISPR-enabled base-editing of *RUBY* – Colony PCR.
Colony PCR will be used to confirm the cloning of sgRNA into pLR3850. Students will visually check the bacterial colonies in the LB plates spread with transfected *E coli* on Day 1. Formation of white colonies suggests successful cloning of the sgRNA into the T-DNA vector. Then, students will perform colony PCR according to Protocol #2. After PCR, save the PCR products for gel electrophoresis on Day 5. For experimental details, see Protocol #2.

Day 5 / Theme #5: Summary and Q&A

Day 5-Morning (9:30-12:00)

Experiment 1.5 & 2.4 (9:30-10:45am): CRISPR/Cas9 mutagenesis of *GFP* & base-editing of *Ruby* – Gel electrophoresis
PCR products from Exp 1.4 and Exp 2.3 will be loaded into the wells of a 1% agarose gel, which will be subjected to gel electrophoresis for ~45 min. The gel will be examined with a UV transilluminator and a gel image will be taken with a gel imaging system. More details of the experiment can be found in protocol #3.

Experiment 2.5 (10:45-11:00am): CRISPR-enabled base-editing of *RUBY* – Visual assessment of RUBY expression
N. benthamiana leaves infiltrated with agrobacterial cells in Experiment 2.1 will be checked and photographed. Leaf areas agroinfiltrated wild-type *RUBY* should display dark red color, Leaf areas agroinfiltrated with *RUBYm2* should not have color change, while leaf areas agroinfiltrated *RUBYm2+ABE#2* should display red color.

Experiment 1.6 & 3.2 (11:00am-12:00): CRISPR/Cas9 mutagenesis of GFP – Microscopy
An epifluorescent microscope can be used to examine the levels of GFP in leaf areas of *N. benthamiana* 16c plants agroinfiltrated with (i) *Cas9-sgRNA/pDGE347* + *TCV-CP* or with (ii)*TCV-CP* alone in Experiment 1.1 in Day 1. GFP levels in (i) should be lower compared to that in (ii).

Lunch break

Day 5-Afternoon (2:00-4:00)

Summary and evaluation of the workshop outcomes: Co-PIs will check and evaluate results from the students and hand Participation Certificates to students who have successfully completed the workshop.

Q & A and future development of CRISPR: Co-PIs will lead a discussion on the five-day workshop experience; including successes, problems and future development of CRIPSR technologies. Students will have ample time to ask questions and receive answers from co-PIs.

Group Photographs: A group photo will be taken at the end of the workshop if necessary.
