# Supplementary material for: Seeing is believing: Genome editing made easy with RUBY for educational purposes
Source: Plant Physiol. 2025 Jul 23;198(3):kiaf265. doi: 10.1093/plphys/kiaf265 (PMC12283223; doi:10.1093/plphys/kiaf265)
Supplement: kiaf265_Supplementary_Data [file kiaf265_supplementary_data.zip › Supplementary Video Legends.docx]

Supplementary Data section

Supplementary Video Legends

**Supplementary Video S1.** A short interview featuring students who participated in the 2023 Summer CRISPR Workshop. On July 14, 2023, Ms. Kimbra Cutlip from the University of Maryland College Park interviewed four of the 11 undergraduate attendees. She later edited the video clips, incorporating select photos she captured, to create this brief highlight video.
